# Using the perceptual past to predict the perceptual future influences the perceived present – A novel ERP paradigm

**DOI:** 10.1371/journal.pone.0237663

**Published:** 2020-09-01

**Authors:** Ellen Joos, Anne Giersch, Kriti Bhatia, Sven P. Heinrich, Ludger Tebartz van Elst, Jürgen Kornmeier

**Affiliations:** 1 INSERM U1114, Cognitive Neuropsychology and Pathophysiology of Schizophrenia, University of Strasbourg, Strasbourg, France; 2 Department of Psychiatry and Psychotherapy, Medical Center–University of Freiburg, Freiburg, Germany; 3 Faculty of Medicine, University of Freiburg, Freiburg, Germany; 4 Institute for Frontier Areas of Psychology and Mental Health, Freiburg, Germany; 5 Faculty of Biology, University of Freiburg, Freiburg, Germany; 6 Eye Center, Medical Center–University of Freiburg, Freiburg, Germany; Universita degli Studi di Udine, ITALY

## Abstract

The information available through our senses is noisy, incomplete, and to varying degrees ambiguous. The perceptual system must create stable and reliable percepts out of this restricted information. It solves this perceptual inference problem by integrating memories of previous percepts and making predictions about the perceptual future.

Using ambiguous figures and a new experimental approach, we studied whether generating predictions based on regularities in the past affects processing of the present and how this is done. Event-related potentials (ERPs) were measured to investigate whether a highly regular temporal context of either ambiguous or unambiguous stimulus variants differently affects processing of a current stimulus and/or task execution. Further, we tested whether symbolic announcements about the immediate perceptual future can replace the past experience of regularities as a source for making predictions. Both ERP and reaction time varied as a function of stimulus ambiguity in the temporal context of a present stimulus. No such effects were found with symbolic announcements.

Our results indicate that predictions about the future automatically alter processing of the present, even if the predictions are irrelevant for the present percept and task. However, direct experiences of past regularities are necessary for predicting the future whereas symbolic information about the future is not sufficient.

## Introduction

**Fig 1:**
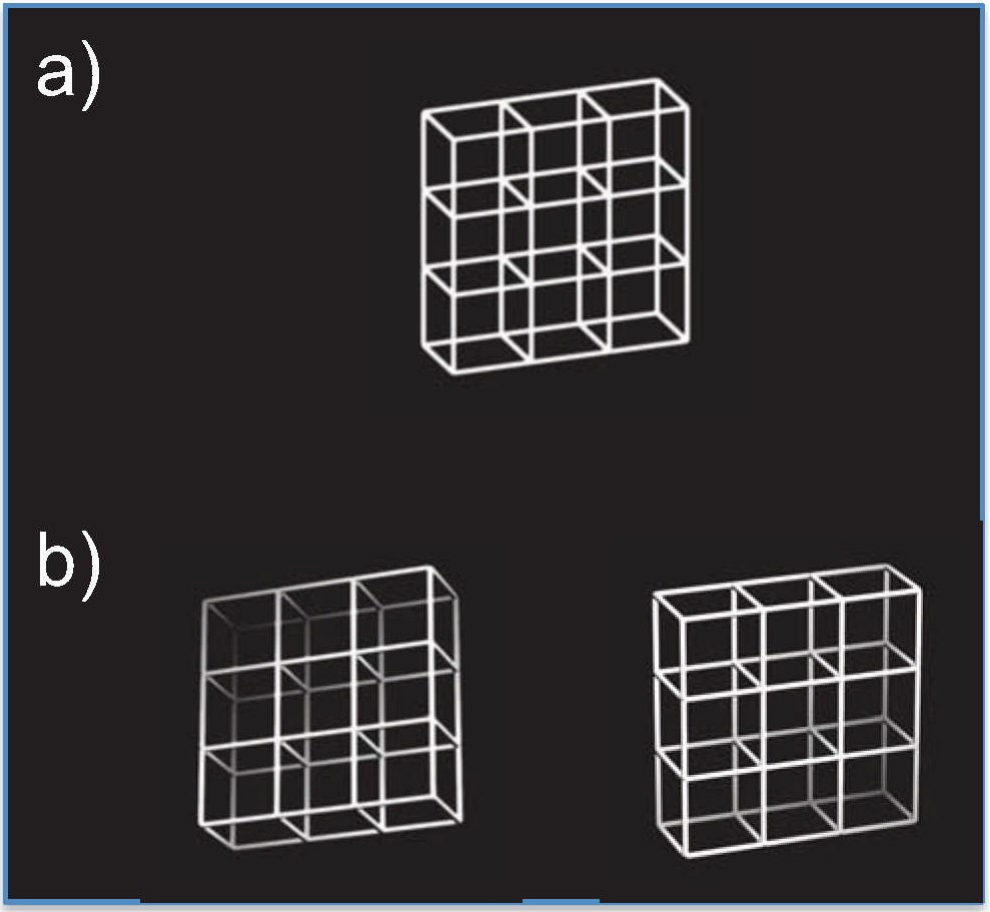
Stimuli. (a) depicts the ambiguous Necker lattice, a variant of the Necker cube [5]. The unambiguous variants are displayed in (b) with the front side facing towards the top (front-top = FT) on the left and the front side facing towards the bottom (front-bottom = FB). Stimuli were created in the laboratory of Dr. Kornmeier.

The information entering our senses is inherently noisy, incomplete, and to varying degrees ambiguous. In order to disambiguate and interpret the strongly limited sensory information, our perceptual system must include non-sensory (top-down) information from spatial and temporal contexts. This enables the brain to construct stable and reliable percepts that allow for a successful interaction with our environment. Perception has already been described as an unconscious inference process by Hermann von Helmholtz [1], where perception results from a combination of bottom-up sensory evidence with top-down contextual information. A more detailed historical overview of the roots of von Helmholtz's "perception as unconscious inference" account can be found in the introduction of Brascamp et al. [2]. One prominent example of the perceptual inference problem is three-dimensional (3D) perception. We live in a three-dimensional world but in the first step of vision, the observed three-dimensional environment is projected onto two-dimensional retinae [e.g. 3]. Therefore, only two of the three dimensions can be accessed directly from this projection. The third dimension, however, has to be reconstructed out of secondary information like occlusion, binocular vision etc. [e.g. 4]. The Necker cube [5] is a famous ambiguous figure consisting of a two-dimensional representation of a three-dimensional cube grid, which can be perceived as two mutually exclusive cube variants with different spatial orientations. Fig 1 presents a so-called Necker lattice, a combination of 9 assembled Necker cubes, together with the two unambiguous lattice variants with 3D cues [6,7]. During prolonged observation, our perception of the Necker cube becomes unstable and alternates between these two interpretations. The reason behind this perceptual instability is that the retinal projection is equally compatible with the two alternative three dimensional cube variants. In fact, the retinal image of the Necker cube is also compatible with other geometric object interpretations with no 90° angles, as nicely demonstrated in Kersten & Yuille (2003) [8]. Nevertheless, our perception typically alternates exclusively between the 90° alternatives. This perceptual bias is already evidence that our perceptual history influences our current percept, since we live in a world where 90° objects are much more common, and thus, more probable than objects that do not contain 90° angles [e.g. 9].

Different lines of research have investigated the influence of the perceptual history, at different time scales, on the current percept. Typical experimental paradigms presented stimuli with different degrees of similarity in sequence and compared the influence of preceding stimuli on the perceptual interpretation of the current stimulus. Several studies reported positive effects (positive priming [10–12], positive hysteresis [9,13], serial dependence [14–16]) of the perceptual history on current percept (e.g. current stimuli are likely to be perceived in the same way as previous ones). In contrast, other studies reported negative effects (e.g. adaptation, negative hysteresis [9,17–19]) of perceptual history on current stimuli (e.g. current stimuli are likely to be perceived as opposite of the previous one).

Taking information of the perceptual past into account is an efficient strategy of the perceptual system to handle the perceptual inference problem. Typically, our environment only slightly changes from one moment to another. Predicting the future using regularities from the immediate past can thus substantially help to overcome the inherent sensory limitations. Concurrently, both speed and efficacy of perceptual processes are increased.

Recent Bayesian probability [8] and predictive coding [20,21] approaches provide a general theoretical framework that may be able to integrate the above mentioned findings. The basic idea is that the perceptual history is used to generate a model of the external world and to make predictions about the upcoming sensory future. A measure of the error between generated predictions and the actual sensory evidence (prediction error) is minimized during a variable number of recurrent loops of feed-forward (bottom-up) and feedback (top-down) neural activity [e.g. 22].

The electroencephalogram (EEG) monitors the activity of the brain non-invasively. Its high temporal resolution allows for observation of neural processing on a millisecond scale. The influence of the immediate perceptual history on the current percept can thus be analysed in terms of EEG correlates. The event-related potential (ERP) is the averaged EEG response over many stimulus repetitions, which isolates processing steps time-locked to the stimulus. Several studies have used ERPs to test predictive coding approaches. In typical paradigms, the predictions are based upon frequent repetitions of the same stimulus in the immediate past. These predictions are then infrequently violated by sudden presentation of a deviant stimulus. Differences between predicted (frequently presented) and unpredicted (rare deviant) stimuli are then interpreted as correlates of the prediction error. The ERP correlate of this postulated prediction error is the so-called Mismatch Negativity (MMN), a negative ERP component between 100 and 250 ms after onset of the deviant stimulus, with maximal amplitude at temporal and frontal electrode locations [MMN, 23–25]. The MMN is regarded as an important physiological correlate within the predictive coding account and is interpreted as reflecting the prediction error, i.e. the outcome of the comparison between prediction and actual sensory input.

The current study differs in two aspects from the previously described MMN studies:

### (1) Stimulus quality instead of stimulus frequency

The stimuli used in the MMN studies introduced above were typically unambiguous, highly visible, and mainly differed in their occurrence frequency. However, in our natural environment, exploiting the perceptual past and relying on a predicted future may become increasingly important in perceptual situations with low quality of the sensory input, e.g. when the stimulus is ambiguous. In this situation, the occurrence frequency of a certain stimulus in a short period of time is less important. Furthermore, stimuli from the immediate past, that are ambiguous or low in visibility, may make predictions about the immediate perceptual future less reliable than unambiguous previous stimuli. Therefore, we presented ambiguous and unambiguous lattice variants [26,27], with ambiguity as the independent stimulus variable to study temporal context effects during perception. The term ‘temporal context’ can refer to differences in temporal aspects of the stimuli such as presentation duration of the stimuli [28,29]. Further, the term is used in memory studies and can refer to features and/or objects that occur simultaneously with an object of interest, which are thus linked together in the perceptual memory [30]. In the current study we present certain sequences of stimuli repeatedly within experimental blocks. This makes an actual stimulus sequence within an experimental block highly memorable and predictable. In the following, the ‘temporal context’ of a certain stimulus thus implied both, the immediately preceding stimulus and the highly predictable subsequent stimulus.

We hypothesize that predictions based on previous experience with an ambiguous stimulus (temporal context = ambiguous) are less reliable than predictions based on previous experience with clear and unambiguous stimuli (temporal context = unambiguous).

### (2) Ambiguity-sensitive ERPs as dependent variables

Kornmeier et al., in a series of ERP studies, presented either ambiguous or unambiguous stimulus variants in separate experimental conditions and compared the resulting ERPs. They found prominent P200 and P400 amplitude effects (Cohen’s d between 0.6 and 1.2). The unambiguous stimulus variants resulted in large amplitudes and the ambiguous stimuli in small amplitudes [26,27]. These prominent ERP effects were found across different categories of stimulus ambiguity (geometry, motion, Gestalt perception) and recently also for smiley stimuli with low and high visibility of their emotional expression [31]. The authors interpreted the P200 and P400 amplitude effects as correlates of certainty about perceptual outcomes [see also 32] or–in other words–as a measure of success in solving the perceptual inference problem. In their recent study they labelled these effects “ERP Uncertainty Effects” [31].

Assuming that this interpretation is valid and that perceptual outcomes are always the result of integrating bottom-up sensory information with top-down temporal context information, it would be reasonable to expect that the sizes of these effects not only depend on the quality of the current stimulus, but also on the quality of the stimuli within the temporal context. The experimental design of our previous studies [26,27,31] did not allow for systematic analyses of sensory quality within the temporal context because the level of ambiguity/visibility was kept constant within conditions (block design).

In contrast to this, in the current study, we presented stimuli in pairs, where stimulus S1 was followed by stimulus S2. Furthermore, we created four different experimental conditions with a paired design (2x2) with differing ambiguity levels of S1 and S2 (ambiguous vs. unambiguous). Designing the experiment in this way allowed us to investigate neural responses elicited by the same S1 stimuli over different levels of ambiguity in its temporal context, i.e. preceding S2 of the previous pair and subsequent S2 of the current pair. We were thus able to investigate neural responses to an ambiguous S1 stimulus and compare an ambiguous temporal context with an unambiguous temporal context. Similarly, neural responses to unambiguous S1 stimuli were compared between ambiguous and unambiguous temporal contexts. We postulate that responses to stimuli S1 should reveal higher ERP amplitudes when the temporal context consists of unambiguous stimuli compared to ambiguous stimuli, meaning that ERP responses should be higher in amplitudes when the temporal context is certain as opposed to uncertain. This effect is expected to be independent of the ambiguity level (ambiguous, unambiguous) of S1 itself. Furthermore, an ambiguous context (i.e. an uncertain temporal context) may drive the observer into an uncertain, and thus, less stable current perceptual state, making them react more hesitantly. As a result of this, we expect longer reaction times.

In short, we hypothesize that the automatic integration of the stimulus information from the temporal context affects processing of the sensory present and the execution of a present task.

The current study consists of two experiments. In Experiment 1, we compared both the reaction times to a stimulus-related task as well as the P200 and P400 ERP components (ERP Uncertainty Effects [26,27,31]) evoked by two factors: ambiguity level of the current stimuli (ambiguous vs. unambiguous) and ambiguity level of the stimuli within the temporal context (ambiguous vs. unambiguous).

In Experiment 1, the temporal context consisted of either ambiguous or unambiguous lattice stimuli. In Experiment 2, we replaced the preceding S2 stimuli with abstract symbolic information about the future stimulus and studied whether this replacement alters the ERP and reaction time results, which were obtained in Experiment 1.

## Material and methods—Experiment 1

### Participants

Thirteen participants (seven females) took part in this study. The median age was 24 with participants ranging from 21 to 34 years old. Twelve participants were right-handed and one was ambidextrous. All participants had normal or corrected-to-normal visual acuity [33] and gave their written informed consent. The study was approved by the ethics committee of the University of Freiburg and was performed in accordance with the ethical standards laid down in the Declaration of Helsinki [34].

#### Stimuli

We used the ambiguous Necker lattice, a combination of nine Necker cubes [5,6] and two unambiguous lattice variants corresponding to the two perceptual interpretations of the ambiguous lattice, see Fig 1. The unambiguous lattice variants included depth cues, like shading, central projection, and aerial perspective based on OpenGL lighting model [35]. The lattice stimuli had a size of 7.5° x 7.5° visual angle. Both ambiguous Necker lattices and unambiguous lattice variants had a mean luminance of 40 cd/m^2^ (the unambiguous stimuli luminance being calculated by averaging the four outer corners of the lattice). All lattices were presented on a black background (0.01 cd/m^2^).

#### Procedure

Participants were tested in a dimly lit room in the Eye Center, in the Medical Center of the University of Freiburg, Germany. They were seated at a distance of 114 cm in front of a Philips GD 402 monochrome CRT screen (refresh rate = 85 Hz, screen resolution = 800×600 pixels), which was operated by an Apple Mac mini computer. During the experiment, participants were instructed to focus their gaze on a fixation point in the middle of the screen.

One observation sequence (OS) consisted of the successive presentation of two lattice stimuli (S1 and S2). Each stimulus was presented for 800 ms. S1 and S2 were temporally separated by an inter-stimulus interval (ISI) of 400 ms. During presentation of the lattice S1, participants were instructed to identify its 3D orientation (front side perceived either right/downwards or left/upwards) and to indicate their percept by key press. During the subsequent presentation of the second lattice (S2), participants compared their perceived 3D orientation of S2 with that of the previously perceived and memorized S1. By pressing separate keys, participants indicated perceived orientation reversal or stability (i.e. percepts of identical 3D orientations of S1 and S2). Key presses were performed on a keyboard with four keys, and key assignment (two scenarios) was counterbalanced between participants.

Key assignment scenario 1: keys 1 and 2 were associated with the orientation task and pressed with the left thumb, with key 1 indicating the left/upwards orientation and key 2 the right/downwards orientation. Keys 3 and 4 were associated with the memory task and pressed with the right thumb, with key 3 indicating perceptual stability and key 4 perceptual reversal trials.

Key assignment scenario 2: keys 1 and 2 were associated with the memory task and pressed with the left thumb, with key 1 indicating perceptual stability and key 2 perceptual reversal trials. Keys 3 and 4 were associated with the orientation task and pressed with the right thumb, with key 3 indicating the left/upwards orientation and key 4 the right/downwards orientation.

**Fig 2:**
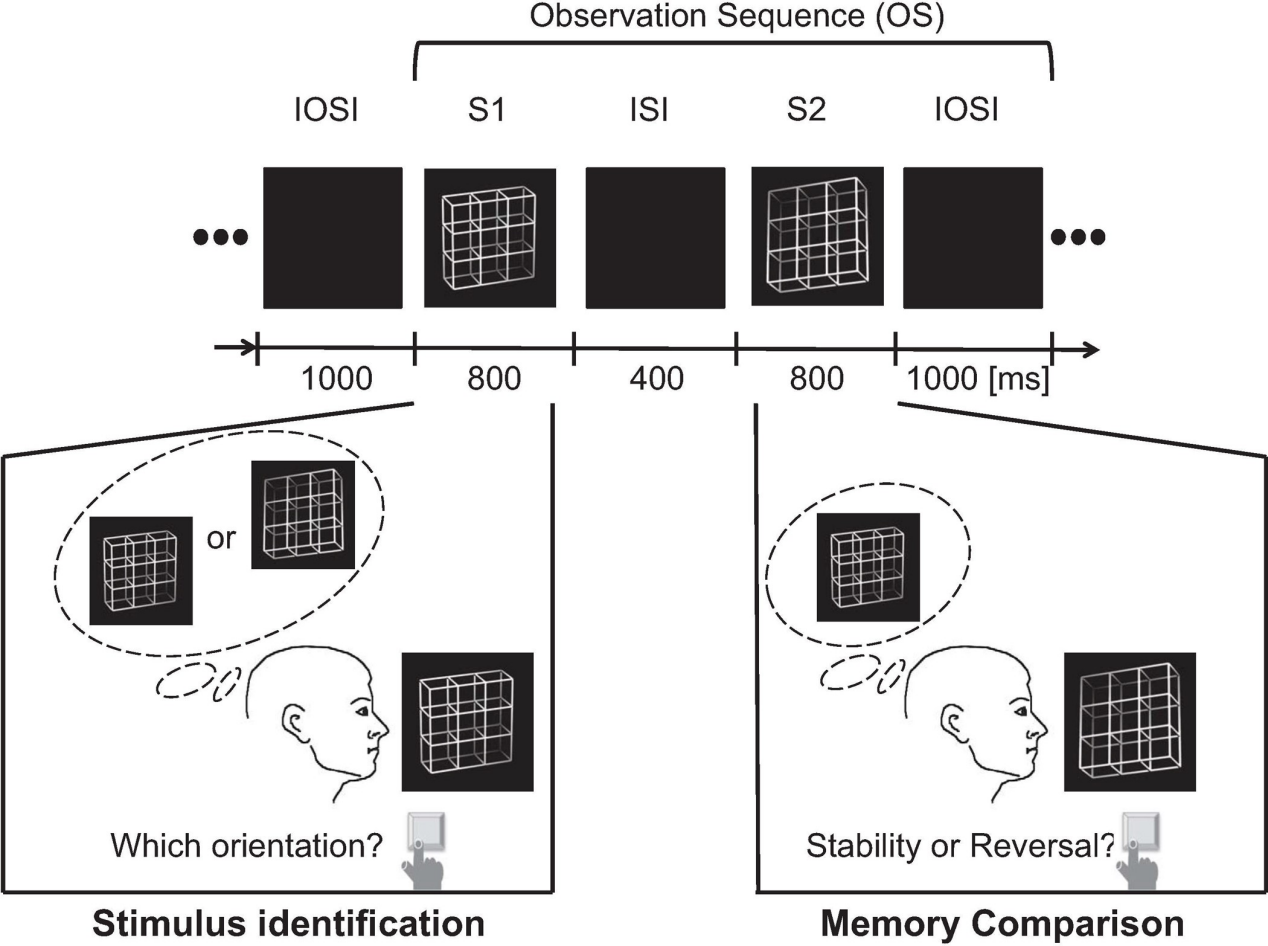
Paradigm of Experiment 1. Stimuli were presented in pairs one after the other and formed one Observation Sequence (OS). Each stimulus was presented for 800 ms. Stimulus 1 (S1) and Stimulus 2 (S2) were presented in succession and temporally separated by an inter-stimulus interval with a dark screen for 400 ms. Presentation of a dark screen for 1000 ms separated subsequent OS from each other. The experimental paradigm consisted of two tasks: during the presentation of the lattice stimulus S1 participants indicated the perceived orientation of S1 (Task 1). During the subsequent presentation of the lattice S2, they compared their perceived S2 orientation with the previously perceived and memorized orientation of lattice S1 and indicated either percepts of identical or reversed orientation (Task 2). Notice that the Task 1 was only related to stimulus S1. Neither the information about the preceding and subsequent stimuli nor information about their ambiguity levels was necessary for the execution of this task.

Successive observation sequences (OS) were separated by an inter-observation sequence interval (IOSI) of 1000 ms (see Fig 2).

**Fig 3:**
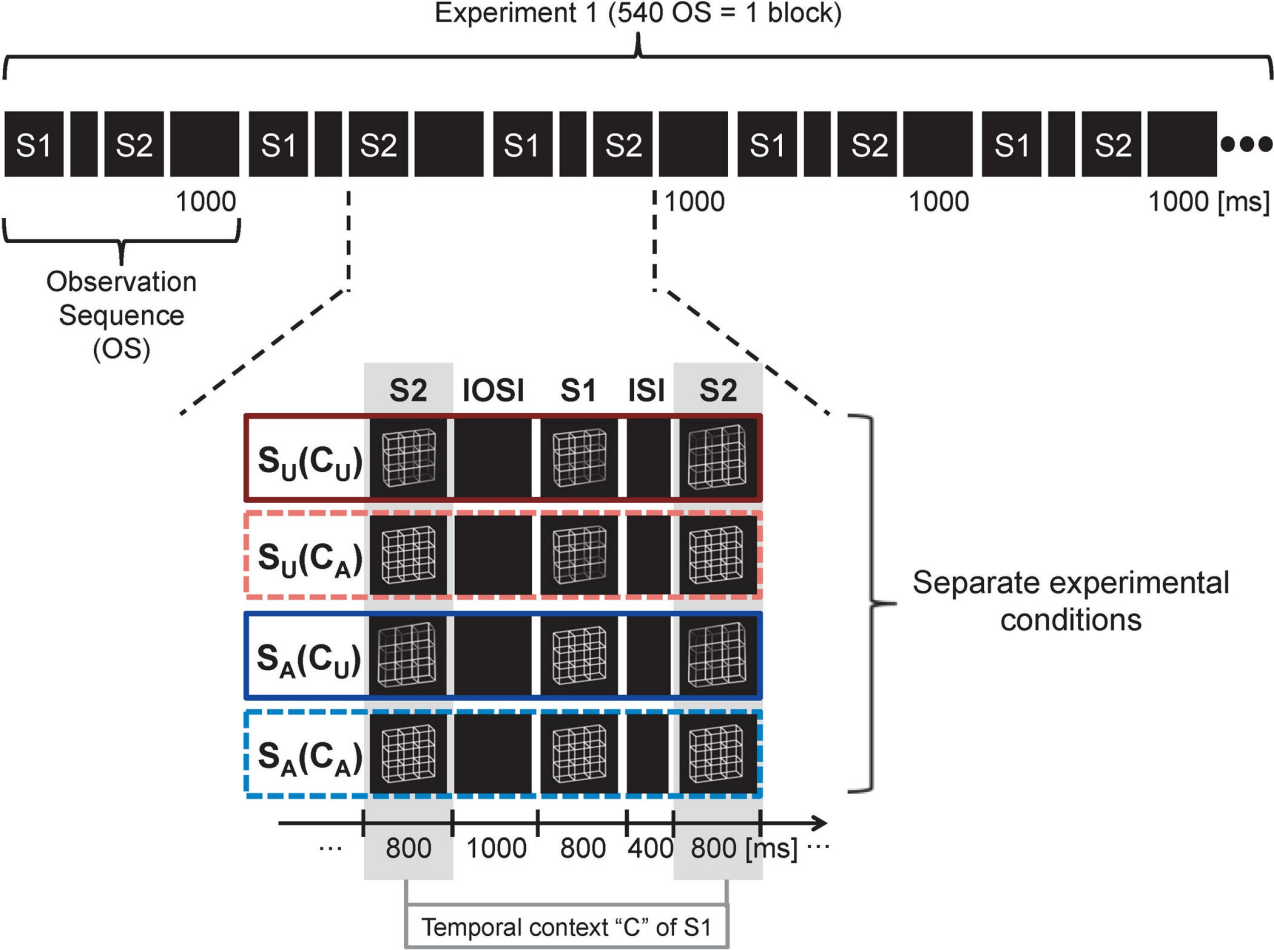
Conditions of Experiment 1. The current experiment consisted of four separate experimental conditions with a 2 x 2 design. S_U_(C_U_): Both lattices S1 and temporal context stimuli (preceding and upcoming S2) were unambiguous; S_U_(C_A_): S1 unambiguous and temporal context ambiguous; S_A_(C_U_): S1 ambiguous and temporal context unambiguous; S_A_(C_A_): both S1 and temporal context ambiguous. Ambiguity levels of S1 and the temporal context were kept constant and were thus highly predictable within conditions but differed between conditions. In Experiment 1, each experimental block consisted of 180 observation sequences (OS) with the same stimulus pairs. Each block was repeated 3 times across the experiment. (U = Unambiguous, A = Ambiguous).

Experiment 1 consisted of four different experimental conditions (see Fig 3). The ambiguity levels of both S1 and S2 varied between but stayed constant within experimental conditions. The analysis only focused on EEG and behavioural responses to stimulus S1 (the currently observed stimulus) as a function of the ambiguity levels of preceding and upcoming stimuli S2 (see details below). S1 stimuli, denoted as "S", occurred in different experimental conditions with two different ambiguity levels with the following coding: S_A_ = ambiguous lattice; S_U_ = unambiguous lattice variant. The stimulus S2 from the preceding pair and the upcoming S2 from the current pair had always the same ambiguity level within an experimental condition. We will label these preceding and upcoming S2 stimuli as the temporal context "C" of S1. The ambiguity level of the temporal context stimuli will be coded as follows: C_A_ = temporal context consists of ambiguous lattices; C_U_ = temporal context consists of unambiguous lattice variants.

The ERP and reaction times to a currently observed stimulus S1 will be described as a function of the ambiguity level of the currently seen stimulus S1 and of its temporal context with the following labels:

S_A_(C_A_): response (i.e. ERPs and reaction times) to an ambiguous S_A_ (S1 from a stimulus pair) as a function of an ambiguous temporal context C_A_ (i.e. ambiguous S2 from the preceding stimulus pair and ambiguous upcoming S2 from the current pair)

S_A_(C_U_): response to an ambiguous S_A_ as a function of an unambiguous temporal context C_U_

S_U_(C_A_): response to an unambiguous S_U_ as a function of an ambiguous temporal context C_A_

S_U_(C_U_): response to an unambiguous S_U_ as a function of an unambiguous temporal context C_U_

In conditions with ambiguous lattice stimuli S_A_, the perceived 3D orientation could reverse endogenously from one stimulus presentation to the next, while the stimulus itself stayed unchanged. The short presentation time of 800 ms prevented perceptual reversals during stimulus presentations. Therefore, perceptual reversals only took place from one presented stimulus to the next.

In conditions with unambiguous stimuli S_U_, the two lattices variants corresponding to the two perceptual alternatives of the ambiguous lattice were presented with a 50% occurrence probability.

Each of the four experimental conditions was subdivided into three shorter experimental blocks that alternated in a pseudo-random order across the experiment. Experimental blocks lasted for about 9 minutes. It is important to note, that the blocked design with multiple repetitions of identical stimulus pairs introduced a sensory regularity within blocks and conditions and made upcoming ambiguity levels of stimuli highly predictable. For example, in the experimental condition S_A_(C_U_), a currently presented S_A_ was always followed by a highly predictable unambiguous S2 and preceded by an unambiguous S2 from the previous stimulus pair, forming an unambiguous temporal context C_U_ of this S_A_.

Participants learned the tasks with the S_U_(C_U_) condition before the EEG experiment in training blocks of four minutes. The training blocks were repeated as many times as needed to reach an error rate of maximal 5% within one block. As a result, the number of repetitions varied slightly between participants.

### Behavioural analysis

#### Lattice orientation

The Necker lattice stimuli used in this experiment can be described in terms of their ambiguity level, as well as in their perceived orientations. Ambiguous and unambiguous variants of the Necker lattice stimuli could be perceived with their front side facing towards the bottom (front-bottom = FB) or towards the top (front-top = FT). We report the ratio between the two perceived orientations of stimulus S1 separately for the four experimental conditions.

**Reversal rates.** We analysed the reversal rates from S2 of the preceding pair to the currently seen S1, i.e. those responses that indicate differently perceived orientation of S2 compared to S1. We separately calculated reversal rates towards percept FB (RR_FT = >FB_) and reversal rates towards percept FT (RR_FB = >FT_) as follows:

$${RR}_{FT=>\;FB}=\frac{\#(S2=FT,S1=FB)}{\#(S2=FT,S1=FB)+\#(S2=FB,S1=FB)}$$

$${RR}_{FB=>FT}=\frac{\#(S2=FB,S1=FT)}{\#(S2=FB,S1=FT)+\#(S2=FT,S1=FT)}$$

Wilcoxon signed-rank tests were used to test for difference of reversal rates between the different directions (RR_FT = >FB_ and RR_FB = >FT_) of reversals. This was done separately for each experimental condition (S_A_(C_A_), S_A_(C_U_), S_U_(C_A_), S_U_(C_U_)). Furthermore, Wilcoxon signed-rank tests were used to compare differences of reversal rates between experimental conditions.

### Effects of sensory quality within the temporal context

#### Reaction time

Reaction times from Task 1 (indicating perceived 3D orientation of lattice S1) were measured from the onset of lattice S1 to the participant’s response. Responses were regarded as physiologically plausible if their earliest occurrence was 150 ms after stimulus onset. Reaction times were valid until the end of the inter-stimulus interval, i.e. 1200 ms after stimulus onset.

#### Electrophysiological recordings

*EEG recordings and pre-processing*. EEG was recorded with 32 active silver/silver chloride electrodes (Brain Products GmbH, 82205 Gilching, Germany) according to the extended 10–20 system [36]. Impedance was kept below 10 kΩ across all electrodes. EEG data were digitized with a sampling rate of 500 Hz, offline digitally filtered with a low-pass at 25 Hz and re-referenced to linked-ears. Data analysis was executed in Igor Pro 6.3 (WaveMetrics, Inc. 10200 SW Nimbus, G-7 Portland, OR 97223, USA).

Blinks and eye movements were detected and trials were excluded from analysis when reaching an artefact threshold of ±100 μV. Amplitudes were measured relative to the baseline, which was defined as the average from 60 ms before stimulus onset to 40 ms after. This baseline was determined following our lab’s previous studies [26,27,31]. The present analysis focused on ERPs evoked by stimulus S1. EEG data from S1 were sorted with respect to the ambiguity levels of the S1 stimuli as well as the ambiguity level of the temporal context stimuli S2. The data were averaged separately for each participant and for each EEG electrode using the onset of S1 as time reference.

*ERP analysis*. Based on results from previous studies, we focused our analysis on two positive ERP components, a P200 with a latency of about 200 ms and fronto-central scalp distribution, and a P400 about 200 ms later with a centro-parietal scalp distribution from the so-called “ERP Uncertainty Effects” [26,27,31]. We selected electrode Cz as the spatial region of interest (ROI). Corresponding temporal ROIs ranged from 100 to 300 ms, covering the latency of the P200 ERP component and from 300 to 600 ms, covering the latency of the P400. We identified the individual peak amplitudes in the temporal and spatial ROIs and measured the average voltage in a ±30 ms time window around the individual peak [37].

We tested for the assumption of normality using the Shapiro-Wilk test. Significant departures from normality were found for the P200 in condition S_A_(C_A_) (W(13) = 0.81, *p* = 0.008) and for the P400 in condition S_U_(C_A_) (W(13) = 0.84, *p* = 0.02). Therefore, we based our statistical analysis of the ERP components on the non-parametric Wilcoxon signed rank test. The Wilcoxon tests were conducted for the P200 and the P400 amplitudes with a predefined alpha of 0.05.

#### Statistical analysis of reaction time and ERP data

The median reaction times and the P200 and P400 data were sorted with respect to the ambiguity levels of S1 and to the ambiguity level of its temporal context.

We tested for the influence of sensory quality within the temporal context on those variables (reaction time, P200, P400) in the case of an ambiguous current stimulus S1 (main effect 1), by comparing responses to condition S_A_(C_A_) with responses to condition S_A_(C_U_). Similarly, we tested this in the case of an unambiguous current stimulus S1 (main effect 2), by comparing condition S_U_(C_A_) with condition S_U_(C_U_). To test for possible differences in effects of sensory quality within the temporal context between ambiguous (S_A_) and unambiguous (S_U_) currently observed stimuli, we calculated the individual differences between conditions S_A_(C_U_) minus S_A_(C_A_) and between conditions S_U_(C_U_) minus S_U_(C_A_) and compared them.

It is important to note that we analysed the amplitudes of P200 and P400 ERP components evoked by lattice stimulus S1. The ERP amplitudes evoked by one and the same stimulus S1, as well as the reaction times of the task, was compared between the two conditions. In one of the conditions, the temporal context stimuli were ambiguous and in the other, the temporal context stimuli were unambiguous.

All Wilcoxon tests reported until now (reversal rates, reaction times, ERP data) were corrected for multiple testing according to the Holm procedure [38]. The effect size *r*_*effect size*_ *(r*_*es*_*)* was calculated by dividing the *Z*-score by the square root of the total number of observations [39].

### Correlation between EEG data, reaction time data, and reversal rates

We calculated Pearson correlation coefficients *r*_*Pearson*_ between the EEG data (P200 and P400 amplitudes), the reaction time data, and the reversal rates. We calculated these correlations on non-normalized and on normalized values. Normalization was done to account for individual differences regarding EEG data (anatomical differences) but also for individual response strategies, which could possibly influence reversal rates and reaction times. Normalization was accomplished within participants by dividing the individual value (e.g. participant 1, P200 peak amplitude, condition S_A_(C_A_)) by the sum of all experimental conditions (e.g. participant 1, P200 peak amplitude of S_A_(C_A_)+ S_A_(C_U_)+ S_U_(C_A_)+ S_U_(C_U_)). We did not correct the resulting p-values of this exploratory analysis for multiple testing.

## Results from Experiment 1

In the present study, we focused on the P200 and P400 components of the ERP Uncertainty Effects [26,27,31] to test whether processing of a currently observed stimulus is affected by the ambiguity levels (ambiguous vs. unambiguous) of stimuli in its temporal context "C" (immediately preceding and upcoming stimuli) and how this is done.

### Behavioural analysis

#### Trial numbers

In Task 1 related to stimulus S1, participants were instructed to indicate the orientation of the currently perceived Necker lattice stimulus. When presented with the currently seen unambiguous stimuli (conditions S_U_(C_U_) and S_U_(C_A_)), participants, on average, responded correctly more than 90% of the time (96.5% ±0.04 SD and 90.9% ±0.12 SD, respectively). When presented with currently seen ambiguous stimuli (conditions S_A_(C_U_) and S_A_(C_A_)), only one stimulus variant was presented so correctness of the response could not be determined.

We restricted the time window for valid responses for all experimental conditions from 150 to 1200 ms after stimulus onset. Participants reacted to this time window almost perfectly and we only had to exclude 0.018% of all trials (0.04% SD) per participant and condition due to invalid response times. Invalid trials are defined as trials containing incorrect responses, responses outside of the predefined time-window and trials containing EEG artefacts. All other trials are defined as valid trials. The average number of valid trials can be found in Table 1 (middle column) and the average number of all stimulus presentations (including EEG artefacts, incorrect responses and responses outside of the predefined time-window) can be found in the right column of Table 1.

**Table 1:** Number of trials of Experiment 1.

	Average number of valid trials (±SD)	Average number of all stimulus presentations (±SD)
S_U_(C_U_)	404 (±71)	537 (±12)
S_U_(C_A_)	358 (±75)	539 (±9)
S_A_(C_U_)	390 (±99)	520 (±53)
S_A_(C_A_)	341 (±100)	492 (±39)

Table 1 displays the average number of valid trials (±SD) across participants in the middle column and the average number of all stimulus presentations (±SD) in the right column, separately for the experimental conditions (rows: U = Unambiguous, A = Ambiguous).

#### Lattice orientation

For the unambiguous stimuli S1 (= S_U_), the ratio of perceived orientations (front-bottom (FB) vs. front-top (FT) view) was averaged across participants. In condition S_U_(C_U_), the ratio was 193:210 and in the condition S_U_(C_A_), the ratio was 176:182. The two stimulus variants were presented with equal frequency by the stimulus program. The deviations of the perceived lattice orientation from exactly equal presentation frequencies are due to some trials being categorised as invalid trials (incorrect responses, responses outside of the predefined time-window, trials containing EEG artefacts). The ambiguous Necker lattices can be perceived in two different orientations. It is known from the literature (e.g. [40]) that observers show a perceptual bias in favour of the front-bottom view (which is a from-above perspective). This a priori bias can also be seen in the results of the current study, in conditions where an ambiguous Necker lattice is the stimulus S1 (S_A_). For the condition S_A_(C_U_), a front-bottom to front-top ratio of 247:142 was observed and for the condition S_A_(C_A_), the front-bottom to front-top ratio was 233:108. The ratios reported are averages across participants.

#### Reversal rates

**Fig 4:**
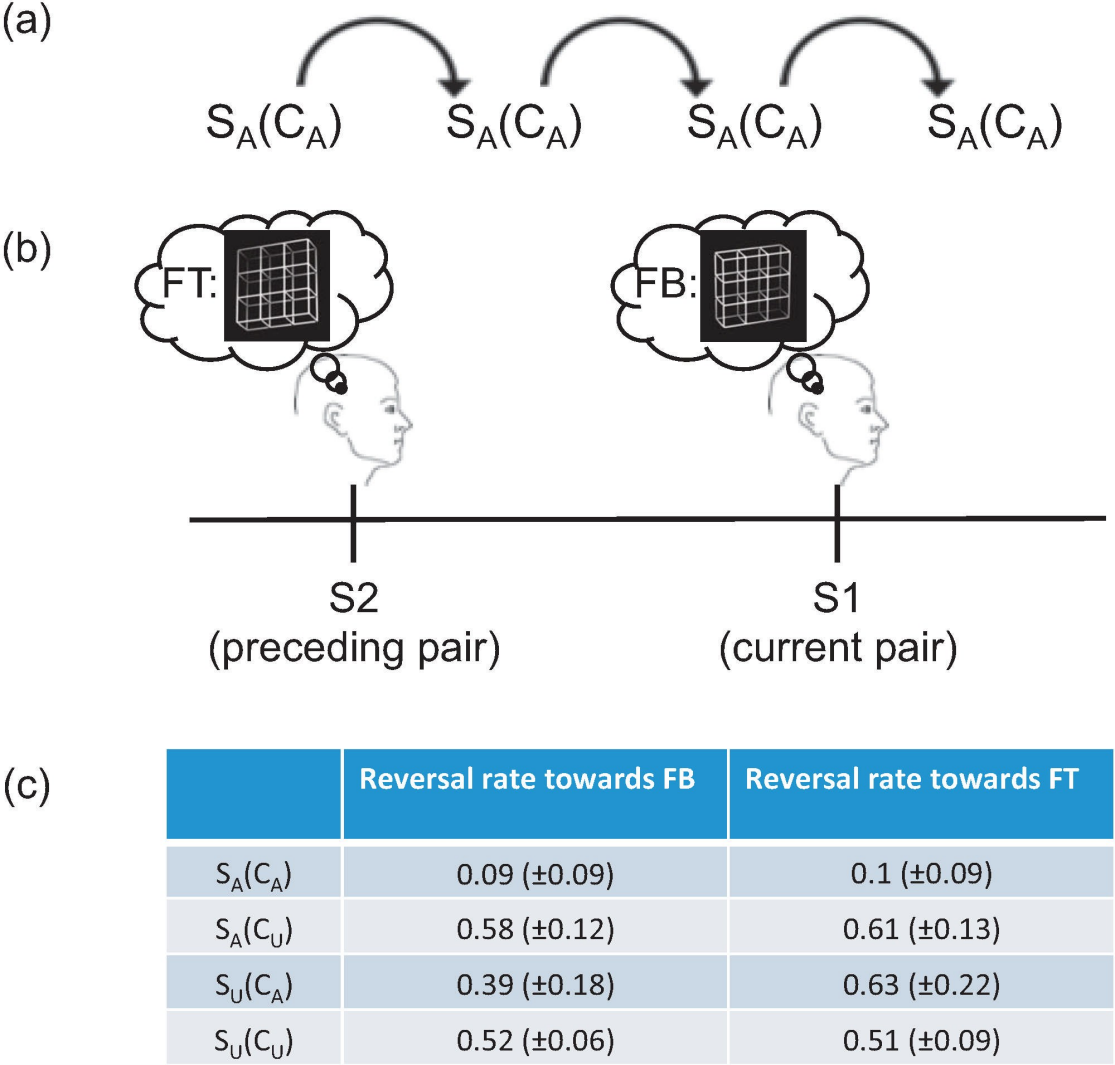
Reversal rate Necker lattices. We calculated the reversal rate towards front-bottom (FB) and the reversal rate towards front-top (FT) views of the Necker lattice from the preceding S2 of the previous pair towards the currently seen S1. A schematic overview of condition S_A_(C_A_) can be seen in a) and an example with stimuli in b). In c) the average (±SD) values are displayed for both view orientations and for each condition.

Numbers of perceptual reversals from S2 of the preceding pair to the currently seen S1 are listed in Fig 4C separately for the four different conditions. Comparing the different directions of reversal (FB = >FT vs. FT = >FB) within conditions, we only found significantly more reversals from FB = >FT compared to FT = >FB in condition S_U_(C_A_) (*Z* = -2.41, *r*_*es*_ = -0.47, *p* = 0.03).

For ambiguous S1 stimuli (S_A_) we did not find differences between the directions of reversals. When comparing reversal rates between conditions S_A_(C_A_) and S_A_(C_U_), we calculated cumulative reversal rates across reversal directions and found significant differences between the conditions (*Z* = -3.18, *r*_*es*_ = -0.62, *p* = 2e-07). We found no significant difference of reversal rates between conditions S_U_(C_A_) and S_U_(C_U_), which were separately analysed for both reversal directions.

We do not see a consistent pattern in the reversal rate results and therefore, FB and FT percepts will not be separately analysed from now on. The EEG and the reaction time data will not be separated depending on their perceptual reversals or stability from a preceding S2 to the currently seen S1. Correlation coefficients between reversal rates, reaction time data and ERP data will be provided after presentation of the main results.

### Effects of sensory quality within the temporal context

In this results section, we will present the analysis of the influence of sensory quality within the temporal context, i.e. ambiguity level of preceding and subsequent S2 stimuli, on a currently seen S1 stimulus. The temporal context was either ambiguous (= C_A_) or unambiguous (= C_U_), see methods section for more detail. Effects of sensory quality within the temporal context were analysed separately for ambiguous S1 stimuli (= S_A_) and for unambiguous S1 stimuli (= S_U_). Further, the interactions between effects of sensory quality within the temporal context of S_A_ and S_U_ conditions were tested. This procedure is adopted for both the reaction time and the ERP data.

Note that all main effects reported hereinafter represent differences in processing of one and the same stimulus information but varying stimulus information in the temporal contexts. We want to particularly emphasize that the information about the temporal context was completely irrelevant for the execution of Task 1 related to S1.

#### Reaction times

**Fig 5:**
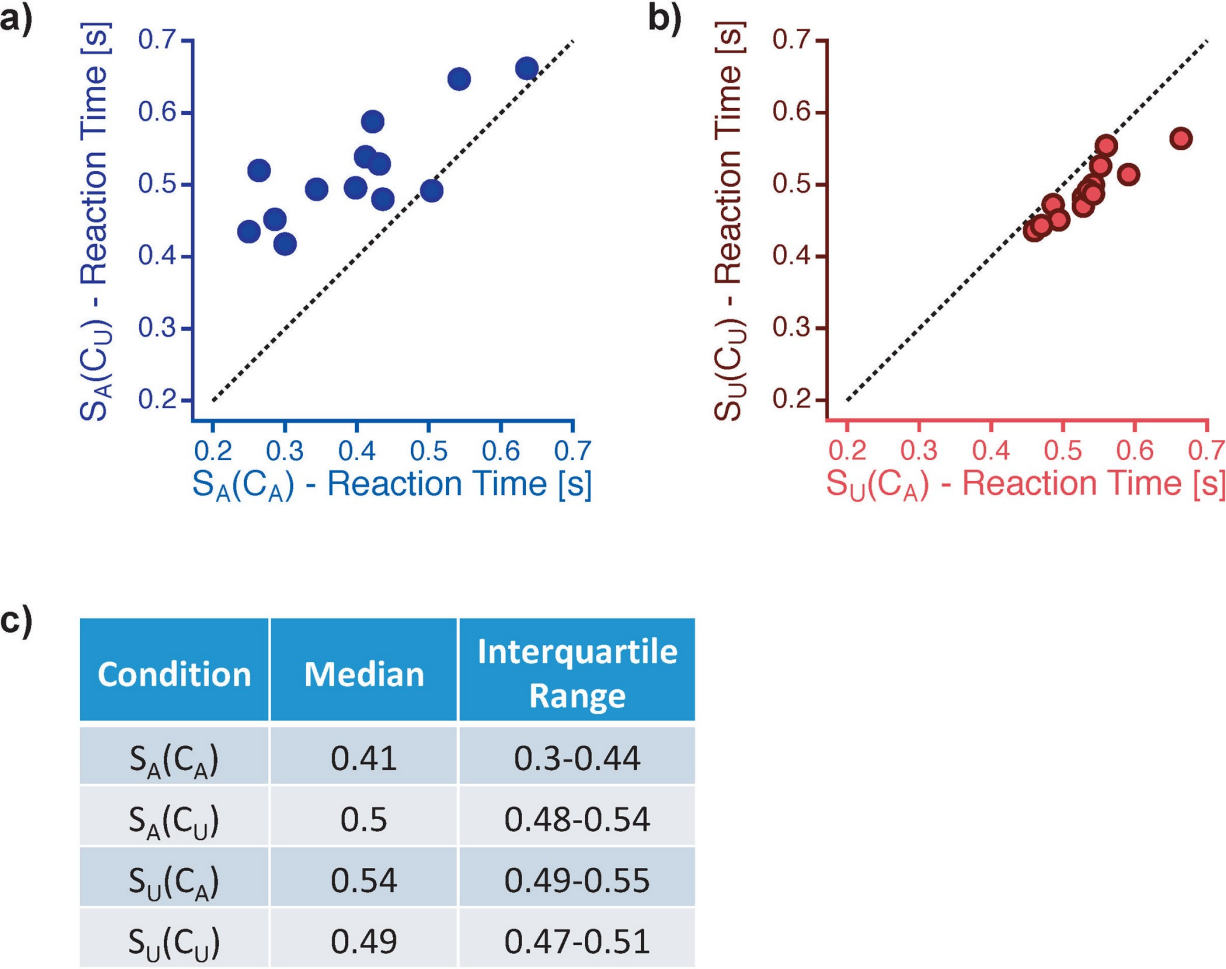
Reaction time data for task 1. Blue colours indicate reaction times to ambiguous stimuli S_A_ (a) and red colours to unambiguous stimuli S_U_ (b). Reaction times show opposite effects of stimulus ambiguity within the temporal context for ambiguous compared to unambiguous currently observed stimuli: reaction times were generally shorter when the stimuli S2 from the temporal context were of the same ambiguity level as the currently perceived stimulus compared to those conditions with differing ambiguity levels of temporal context stimuli S2 compared to the perceived S1. (c) List of median reaction time [s] values with the interquartile ranges [s], separately for each experimental condition.

Reaction times of Task 1 related to an ambiguous stimulus S_A_ were longer if the temporal context was unambiguous compared to an ambiguous temporal context (S_A_(C_U_) vs. S_A_(C_A_): *Z* = -3.11, *r*_*es*_ = -0.61, *p* = 0.00024). This reaction time effect can be seen in the scatter plots in Fig 5A (12 out of 13 data points are above the diagonal).

We found the opposite effect if the observed stimulus was unambiguous. Reaction times of Task 1 related to an unambiguous stimulus S_U_ were shorter if the temporal context was unambiguous compared to an ambiguous temporal context (S_U_(C_U_) vs. S_U_(C_A_): *Z* = -3.18, *r*_*es*_ = -0.62, *p* = 0.00012). This effect can be seen in the scatter plots in Fig 5B (all data points are below the diagonal). Median values and interquartile ranges of reaction times for all experimental conditions can be found in Fig 5C.

The differences in reaction time context effects is statistically indicated by an interaction: Comparing the reaction time differences (S_A_(C_A_)–S_A_(C_U_)) with (S_U_(C_A_)–S_U_(C_U_)) reveals a significant effect (*Z* = -3.18, *r*_*es*_ = -0.62, *p* = 0.00012).

### ERP data

**Fig 6:**
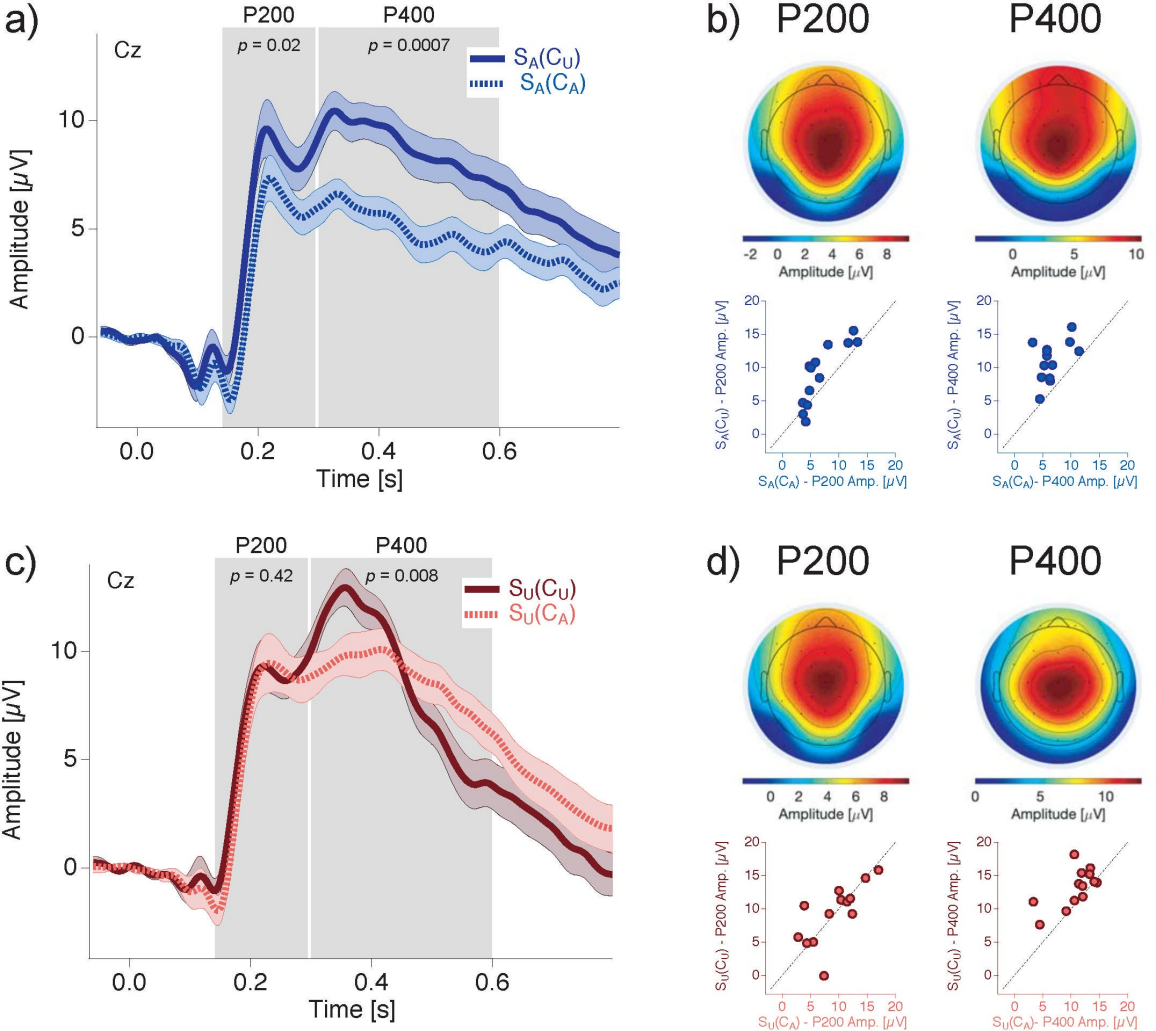
ERP effects of sensory quality within the temporal context. (a) ERP traces at electrode Cz during perception of an ambiguous lattice S_A_, when the stimuli in the temporal context (i.e. S2 from the preceding pair and the predicted S2 from the current pair) were unambiguous (“S_A_(C_U_)”, dark blue continuous trace) and when the stimuli from the temporal context were ambiguous (“S_A_(C_A_)”, light blue dashed trace). Notice that the same ambiguous current S_A_ lattice stimulus evoked larger P200 and P400 amplitudes with an unambiguous temporal context compared to an ambiguous temporal context. (b) Voltage maps (top) showing the spatial distribution of P200 (left, t = 214 ms) and P400 (right, t = 326 ms) and scatter plots (bottom) showing individual mean amplitude data for the P200 (left) and the P400 (right), which correspond to (a). Notice that for almost all participants the P200 and P400 ERP components evoked by S_A_ show larger amplitudes when the temporal context stimuli were unambiguous (data points are above the diagonal). (c) ERP traces during perception of an unambiguous lattice S_U_, when the temporal context stimuli were unambiguous (“S_U_(C_U_)”, dark red continuous trace) and when the temporal context stimuli were ambiguous (“S_U_(C_A_)”, light red dashed trace). Notice that the amplitude of the P400 evoked by the one and the same unambiguous present S_U_ lattice stimulus varied as a function of the ambiguity level within the temporal context. No such effect is visible for the P200. (d) Same logic as in (c) but with an unambiguous present stimulus S_U_ (data related to c; Voltage maps: P200—t = 222 ms, P400—t = 358 ms). U = unambiguous, A = Ambiguous, S = stimulus S1, C = temporal context (preceding and subsequent S2).

Fig 6A displays the ERP traces at electrode Cz evoked by an ambiguous currently observed stimulus, separately for condition S_A_(C_A_), in a block in which the temporal context was ambiguous (light blue dotted trace) and for condition S_A_(C_U_), in a block in which the temporal context was unambiguous (dark blue traces). The amplitudes of both the P200 and the P400 were significantly larger in the case of an unambiguous temporal context compared to an ambiguous temporal context (S_A_(C_U_) vs. S_A_(C_A_): P200: *Z* = -2.3, *r*_*es*_ = -0.46, *p* = 0.02; P400: *Z* = -3.18, *r*_*es*_ = -0.62, *p* = 0.0007). Fig 6A shows this effect in the grand mean ERP traces (electrode Cz) and Fig 6B left shows the individual data in scatter plots. In the left scatter plot, each point represents the P200 amplitudes evoked by the ambiguous currently observed stimulus S_A_ from one individual participant when the temporal context is also ambiguous (S_A_(C_A_): abscissa), versus an unambiguous temporal context (S_A_(C_U_): ordinate). The data points for the most participants (only three exceptions) are located above the diagonal, confirming the above-described temporal context effect on the P200 amplitude. The corresponding context effect for the P400 amplitude moves in the same direction and is even larger than the P200. This is visible in all participants, as indicated in the corresponding scatter plot (Fig 6B right).

Fig 6C displays the ERP traces at electrode Cz for an unambiguous current lattice S_U_, separating the ambiguous temporal context C_A_ (light red dotted trace) from the unambiguous temporal context C_U_ (dark red traces).

In contrast with the findings from an ambiguous current stimulus S_A_, the P200 did not show an effect of stimulus ambiguity within the temporal context when the current stimulus was unambiguous S_U_ (S_U_(C_U_) vs. S_U_(C_A_): P200: *Z* = -0.25, *r*_*es*_ = -0.05, *p* = 0.42). The corresponding scatter plot (Fig 6D left) shows that six out of 13 points are above the diagonal but seven points are below.

Consistent with the findings from an ambiguous current stimulus S_A_, the amplitude of the P400 evoked by an unambiguous current stimulus S_U_ was significantly larger when the temporal context was unambiguous than when it was ambiguous (S_U_(C_U_) vs. S_U_(C_A_): P400: *Z* = -2.76, *r*_*es*_ = -0.54, *p* = 0.0085). This grand mean effect (Fig 6C) can be seen in more detail in the scatter plot in Fig 6D right, where 11 out of 13 points are located above the diagonal, confirming the above-described amplitude difference between conditions.

The P200 results indicate a significant interaction between effects of stimulus ambiguity within the temporal context of an ambiguous stimulus S_A_ and an unambiguous stimulus S_U_ (S_A_(C_U_)-S_A_(C_A_) vs. S_U_(C_U_)-S_U_(C_A_): P200: *Z* = -2.41, *r*_*es*_ = -0.47, *p* = 0.02). The P400 was similarly modulated by the stimulus ambiguity within the temporal context and thus no such interaction was found for the P400 (*Z* = -1.71, *r*_*es*_ = -0.34, *p* = 0.09).

### Correlation between EEG data, reaction time data, and reversal rates

We calculated correlations between the EEG data (P200 and P400 amplitudes) and the median reaction times (see Table A in S1 File), between the EEG data and the reversal rates (see Table B in S1 File), as well as between the median reaction times and the reversal rates (see Table C in S1 File). These correlations were calculated separately for each experimental condition. This exploratory post-hoc analysis was not systematically corrected for multiple testing. However, since we calculated 20 independent correlation coefficients in total (not counting the additional tests with the non-normalized data), we pre-defined an alpha threshold of 0.01.

There are no significant results when correlating the EEG data (P200, P400) with the median reaction time, the EEG data (P200, P400) with the reversal rates, and the reversal rates with the reaction time.

## Summary and discussion of Experiment 1

We compared the amplitudes of two ERP components evoked by the same current stimulus and the reaction times of a stimulus-related task in a condition with ambiguous stimuli in the temporal context C_A_ (i.e. an ambiguous preceding and an ambiguous subsequent stimulus) with a condition with unambiguous stimuli in the temporal context C_U_ (i.e. an unambiguous preceding and an unambiguous subsequent stimulus). Each condition consisted of several experimental blocks. Within the blocks, the condition-specific ambiguity levels of the presented stimuli were kept constant, which made the stimulus sequence highly predictable (see Fig 3).

### ERP results

We found that the P400, evoked by the same stimulus, was generally larger, with an unambiguous temporal context compared to an ambiguous temporal context. This effect was observed irrespective of the ambiguity level of the currently observed stimulus.

We found a similar effect for the P200, when the currently observed stimulus was ambiguous. In contrast, we found no such P200-effects when the currently observed stimulus was unambiguous.

### Reaction time results

The ambiguity level of the temporal context stimuli also affected reaction times related to the execution of Task 1. More specifically, reaction times from Task 1 related to the same unambiguous stimulus S_U_ were longer, if the temporal context contained ambiguous compared to unambiguous stimuli. This finding is striking, because Task 1 was exclusively related to the currently observed stimulus, whereas the ambiguity levels of the stimuli in the temporal context were irrelevant for its execution.

We expected a similar reaction time effect of the temporal context when an ambiguous stimulus S_A_ was observed. However, we found an opposite effect with shorter (rather than longer) Task 1-related reaction times with ambiguous context stimuli compared to than unambiguous temporal context stimuli.

### The role of perceptual reversals for the reported ERP and reaction time effects

Our experimental paradigm contained conditions where the level of ambiguity changed between S1 and S2 stimuli (S_A_(C_U_) and S_U_(C_A_)) and conditions where the ambiguity level stayed the same between stimulus presentations (S_A_(C_A_) and S_U_(C_U_)). Our analysis so far suggests that the ERP amplitude and the reaction time effects reflect differences in the ambiguity level of the stimuli in the temporal context (S2) of a currently perceived stimulus (S1).

However, another aspect that changed (or stayed stable) between stimulus presentations is the perceived 3D orientation of the presented lattice stimuli. In the case of the unambiguous lattice stimuli, we presented the stimulus variants with depth cues corresponding to the two most probable perceptual interpretations of the ambiguous lattice variant (see Fig 1) with a predefined rate (of 0.5) of 3D orientation reversals (by the stimulation program) from one stimulus to the next. The perceptual interpretations of the unambiguous stimulus variants, as indicated by the participants, almost fully corresponded to what was presented on the screen (see section “Lattice orientation” in the behavioural results section).

During observation of the ambiguous Necker lattice, the perceptual interpretation is endogenously driven (see [41] for more information about perceptual endogenous reversals during observation of ambiguous figures). As a result, the rate of perceptual reversals could not be controlled by the computer program when the condition included ambiguous lattices (S_A_(C_A_), S_A_(C_U_), S_U_(C_A_)). Consequently, reversal rates between stimuli could vary between the four experimental conditions and such variations could also have contributed to the ERP amplitude and reaction time effects reported above.

We compared reversal rates between the experimental conditions and found significantly reduced reversal rates in condition S_A_(C_A_), i.e. when an ambiguous stimulus S1 was combined with an ambiguous temporal context, compared to the other conditions. In order to study how much the reversal rates influenced the ERP amplitude and reaction time modulations, we calculated post-hoc correlation coefficients between reversal rates, amplitude effects and reaction times, respectively, but found no significant correlation. We thus conclude that differences in reversal rates cannot explain the observed amplitude and the reaction time effects.

### P200 vs. P400 ERP components

The ambiguity level of the stimuli in the temporal context of a currently observed stimulus affects the P400 amplitude, regardless of the currently observed stimulus being ambiguous or unambiguous. The pattern of results is slightly different for the P200 ERP component, where we only see effects of sensory quality within the temporal context if the currently observed stimulus is ambiguous. Those effects cannot be observed when the currently presented stimulus is unambiguous. Potential explanations for this observation will be outlined in the General Discussion.

### Are the effects of sensory quality within the temporal context low-level or high-level/cognitive effects?

A possible explanation for the ERP effects described above could be that unambiguous lattice stimuli are processed differently. The unambiguous lattice stimuli have brighter and darker edges that are cues for the third dimension and result in different local retinotopic adaptation during their observation. The ambiguous lattice stimuli have exclusively isoluminant edges with an intermediate brightness and so, homogeneous adaptation across the retinotopic visual maps could be expected in this case. Thus, a visual stimulus presented to a perceptual system in a differently adapted state may thus be differently processed. Therefore, the ERP components evoked by this stimulus may differ in amplitude as a function of the difference in adaptation levels. Temporal context stimuli differing in their degree of ambiguity may drive the perceptual system into differently adapted states and thus account for the previously reported ERP effects. This low-level interpretation of the results is related to the findings from Cicchini et al [15], where the authors found low-level influences of the immediate past on perception in an uncertain but not in a certain situation.

On the other hand, several results indicate an involvement of high-level cognitive processes. The late latencies of the affected ERP components, 200 ms and 400 ms after stimulus onset, the reaction time modulations, as well as the lack of correlations between ERP data, reversal rates, and reaction times all indicate this.

We suggest that the effects of the ERP amplitude and reaction time are related to differences in the ambiguity level of the temporal context. Several studies have shown that the perceptual system continuously evaluates the sensory regularities from the past to make predictions about the future [e.g. 23,24]. Therefore, we postulate that the previously presented effects may be related to such evaluation and prediction processes.

In a next experimental step, we aimed to estimate at which level a potential evaluation of regularities within the temporal context takes place. It could be that the direct perceptual experience of regularities in the immediate perceptual past is a necessary condition for such predictions about the future. Alternatively, this effect could be located at such a high level that informing the observer about the identity of a future stimulus with an abstract symbol, making the future stimuli 100% predictable, could be sufficient.

The previously found effects should disappear when the information about the perceptual future is only provided in an abstract symbol, if they reflect the predictability of the perceptual future based on the direct perceptual experience of regularities in the temporal context. However, if the abstract information is sufficient, the ERP effects should still be observable.

In Experiment 2, we were unable to measure all four conditions from Experiment 1 due to limited time. Therefore, we focused on the two conditions with an ambiguous current stimulus S1 and ambiguous vs. unambiguous temporal context stimuli S2. The reason behind this being that Experiment 1 revealed amplitude effects for both ERP components, the P200 and the P400. We were interested in how the specific experimental manipulation of Experiment 2 may affect the amplitude effects of both ERP components.

## Experiment 2

In Experiment 2, we investigated whether the same results of Experiment 1 can be found when information to predict the perceptual future is provided in an abstract symbol, and not based on the direct perceptual experience of sensory regularities in the past.

**Fig 7:**
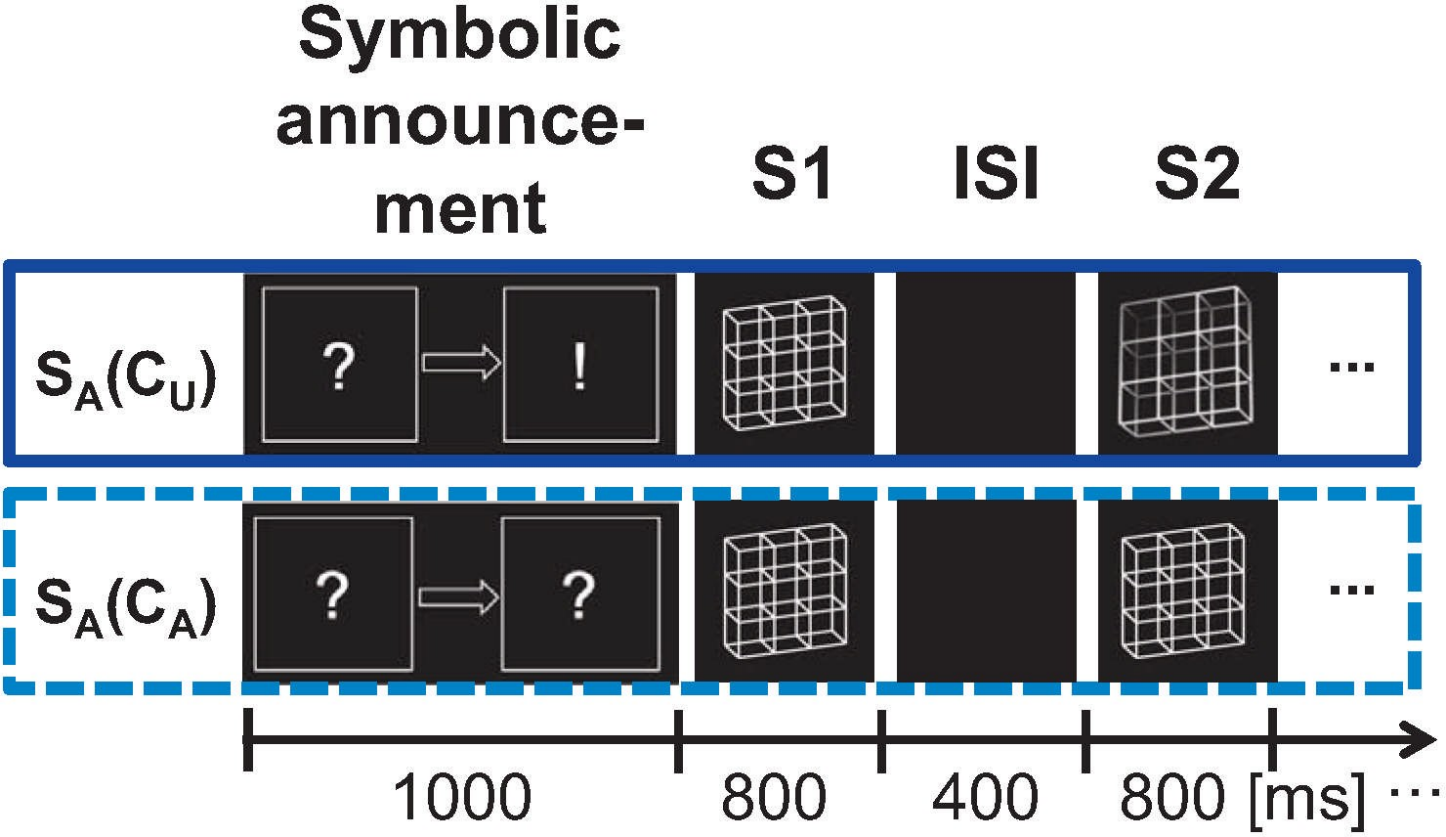
The symbolic announcement in Experiment 2. Ambiguous stimuli are announced by question marks, while unambiguous stimuli are announced by exclamation marks. Before each block of condition S_A_(C_U_) a picture was presented with a question mark symbol (left) pointed towards an exclamation mark symbol (right). Before each block of condition S_A_(C_A_) a picture with two question marks was presented.

**Fig 8:**
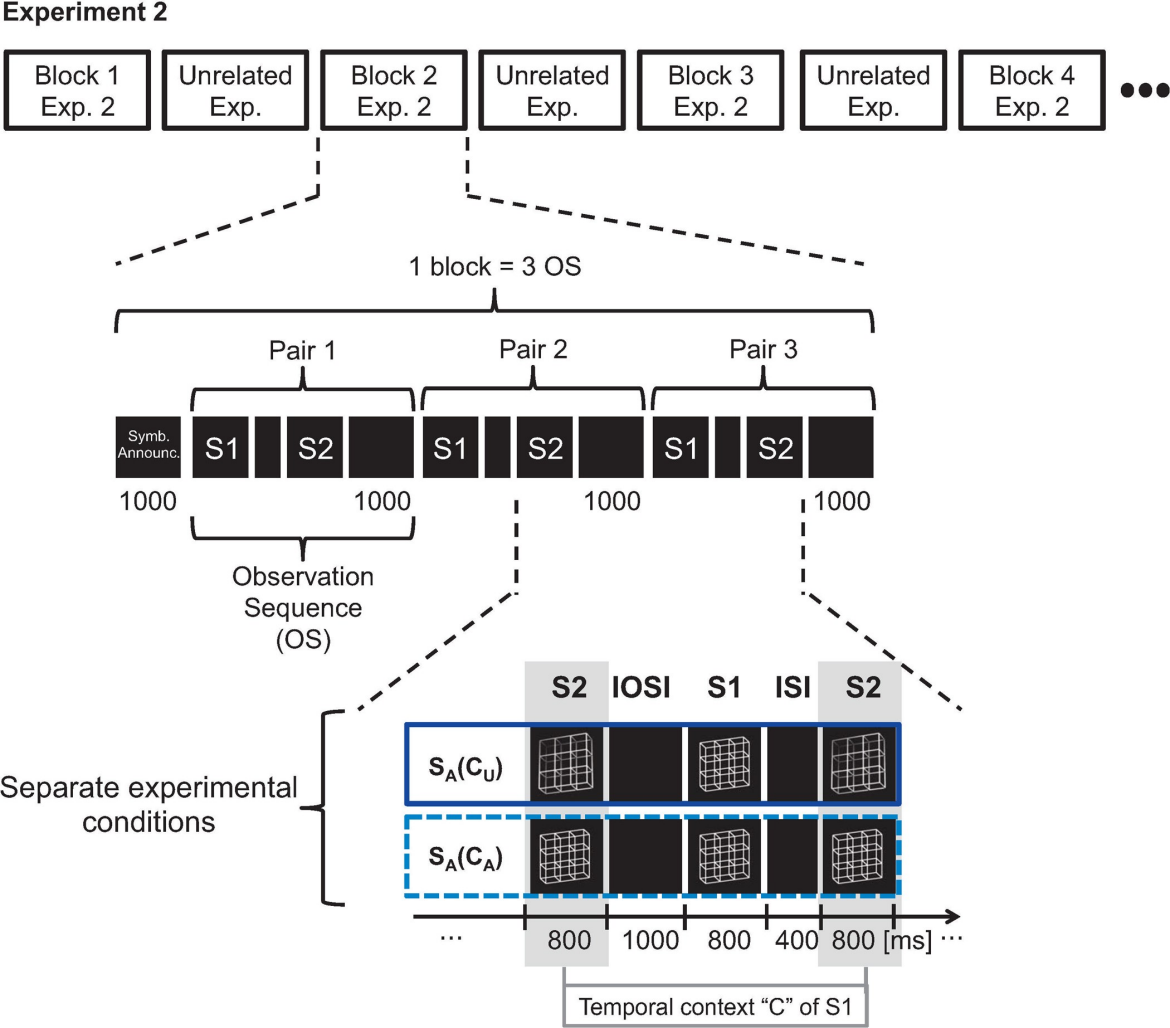
Conditions of Experiment 2. Experiment 2 consisted of two separate experimental conditions (bottom part). Within experimental conditions, ambiguity levels of currently observed S1 stimuli and ambiguity levels of their temporal context (preceding and subsequent S2) were kept constant and were highly predictable within conditions. The ambiguity level of the temporal context differed between conditions. S_A_(C_U_): current ambiguous stimulus S_A_ and unambiguous temporal context; S_A_(C_A_): both S_A_ and temporal context ambiguous. One observation sequence (OS) consisted of an S1 stimulus (800 ms), an inter-stimulus interval (400 ms), a S2 stimulus (800 ms), and an inter-observation sequence interval (1000 ms). One experimental block consisted of a symbolic announcement and three repetitions of the observation sequence. Each block of Exp. 2 was followed by an experimental block from another unrelated experiment with completely different stimuli to increase the temporal distance between blocks of Exp. 2 and thus to minimize low-level memory effects (e.g. priming, adaptation, etc.) from one block to the other. (U = Unambiguous, A = Ambiguous).

Therefore, we changed the paradigm from Experiment 1 in the following way:

(I) Before presenting the stimulus pairs of a specific condition (S_A_(C_A_) or S_A_(C_U_)), we displayed a symbolic representation of the experimental condition that followed. We did not present the actual stimuli that were shown during the experimental condition to avoid low-level effects like adaptation. Instead, two symbols were presented on a screen with one symbol being on the left and the other being on the right. A symbol could be either a question mark, coding for an ambiguous stimulus, or an exclamation mark, coding for an unambiguous stimulus. The position of the symbols on the screen coded for the two stimuli presented in one observation sequence. The left symbol coded for the first stimulus (S1) and the right symbol for the second stimulus (S2; see Fig 7).

(II) We shortened the experimental block durations dramatically from around 9 minutes to 9 seconds. Consequently, each experimental block consisted of only three observation sequences resulting in three repetitions of a specific stimulus pair S1S2 (see Fig 8). This shortening of block duration allowed us to strongly increase the number of blocks. Note that in Experiment 2, the term experimental “block” refers to a presentation of an experimental condition lasting 9 seconds. This includes three repetitions of an observation sequence. Such a short experimental block is immediately followed by the next 9 seconds presentation of an experimental condition (block). The conceptual meaning of an experimental block is identical between Experiment 1 and 2. Only the duration and therefore, the number of repetitions of observation sequences differ between blocks of Experiment 1 and 2.

(III) We aimed to extinguish short-term memory effects from one block of Experiment 2 to the next one by a 'nesting technique'. The blocks of Experiment 2 were interlaced with blocks from a second experiment lasting for about 10 seconds. This second experiment used completely different stimuli and was not related to the lattice stimuli. Therefore, each block of Experiment 2 was followed by a block from this additional experiment with different stimuli.

(IV) Due to limitations in the total experimental time, we only varied the ambiguity levels of the temporal context stimuli (S2) but not of the currently observed stimuli (S1). Meaning that in Experiment 2, only conditions S_A_(C_A_) and S_A_(C_U_) were presented. The sequence of blocks was pseudo-randomized.

The rationale behind these changes in the experimental design from Experiment 1 to Experiment 2 was the following:

The announcement (I) at the beginning of each experimental block contained only abstract symbolic information about the ambiguity levels of the upcoming series of three stimulus pairs. The stimuli themselves were not experienced during this announcement. Additionally, the announcement mostly avoided a surprise-P3b [42–44] ERP response to the very first stimulus (or stimulus pair) within one experimental block. The reduction of block length (II) allowed us to increase the number of experimental blocks for each condition from 3 (Exp. 1) to 120 (Exp. 2). This enabled separate analyses of the effects of sensory quality within the temporal context, reported in Exp. 1, for each of the three stimulus pairs within a block. The interlacing of the unrelated experimental blocks, resulting in an alternation of stimulus types between blocks (III), was intended to allow for the recovery of the perceptual system from possible lower-level conditioning effects (types of serial dependence or adaptation) or recency effects, potentially resulting from the repetitions of one and the same stimulus pair per block.

As a result of this experimental manipulation, the first pair of each experimental block should be unaffected by lower-level footprints from the immediate past. It can only be influenced by the symbolic announcement, assuming that the recovery period introduced by the interspersed unrelated experimental blocks was long enough. However, the second pair was preceded by one presentation of a stimulus pair and the third pair was preceded by two presentations of a stimulus pair. The influence of memory on the three separately analysed stimulus pairs allowed us to study the different effects of cognitive (pair 1) and sensory (pair 2 and pair 3) temporal context information on the processing of a currently observed stimulus. We were also able to study the different effects on related reaction times.

## Material and methods—Experiment 2

### Participants

Twenty-three participants (16 female) took part in Experiment 2. The median age was 24, with participants ranging from 19 to 31 years old. All participants had normal or corrected-to-normal visual acuity [33] and gave their written informed consent. The study was approved by the ethics committee of the University of Freiburg and in accordance with the ethical standards laid down in the Declaration of Helsinki [34]. We had to exclude two participants from the analysis due to low number of trials that survived the artefact rejection (<30 in at least one condition). Eighteen participants were right-handed, two participants were left-handed, and one participant was ambidextrous.

### Procedure

Experiment 2 was very similar to Experiment 1 with three exceptions:

1. At the beginning of each experimental block, we announced the experimental condition (S_A_(C_A_) or S_A_(C_U_)) that the block belonged to abstract symbols (see Fig 7)
2. Experiment 2 was restricted to conditions S_A_(C_A_) and S_A_(C_U_) (see Fig 8). As a result, only the ambiguity level of the stimuli S2 in the temporal context varied, whereas the currently perceived stimulus S1 in the analysis window stayed ambiguous.
3. In this Experiment 2 one experimental block consisted of the symbolic announcement and only three stimulus pairs. With this we decreased the block duration from 9 minutes (Exp. 1) to 9 seconds (Exp. 2) and concurrently increased the number of experimental blocks per condition from 3 (Exp. 1) to 120 (Exp. 2).
4. In this Experiment 2 it was important to extinguish short-term perceptual memory between experimental blocks as much as possible. Thus, we separated the blocks from Experiment 2 by blocks from a separate and unrelated experiment with completely different stimuli (smiley stimuli with different emotional expressions).

Before the start of the main experiment, participants learned the tasks in training blocks of three minutes. In the training blocks, we presented the paradigm with only unambiguous stimuli, which allowed us to distinguish between correct and false responses. The training blocks were repeated as many times as needed to reach a maximal error rate of 10% within one block. Thus, the number of repetitions varied slightly between participants.

### Analyses

Like in Experiment 1, we analysed the ERPs evoked by stimulus S1 from a pair S1S2. In Experiment 2, this S1 stimulus was always ambiguous and is thus labelled accordingly as S_A_ (see Fig 8 for details). The EEG data from this stimulus S_A_ were sorted with respect to participant, to the ambiguity level of the temporal context stimuli S2, to the order of S_A_ in the experimental block (S_A_-pair 1, S_A_-pair 2, S_A_-pair 3), and to electrode. The onset of S_A_ served as a time reference.

As in Experiment 1, we selected electrode Cz as the spatial region of interest (ROI) in Experiment 2. Corresponding temporal ROIs ranged from 100 to 300 ms, covering the latency of the P200 ERP component, and from 300 to 600 ms, covering the latency of the P400. We identified the individual peak amplitudes in the temporal and spatial ROIs and measured the average voltage in a ±30 ms time window around the peak [37].

We tested for the assumption of normality using the Shapiro-Wilk test. Significant departures from normality were found for the P200 in condition S_A_(C_U_)-pair 1 (W(21) = 0.9, *p* = 0.04) and for the P400 in condition S_A_(C_A_)-pair 1 (W(21) = 0.87, *p* = 0.008). Therefore, we based our statistical analysis on the non-parametric Wilcoxon signed rank test.

Reaction times were regarded as physiologically plausible if their earliest occurrence was at least 150 ms after stimulus onset. Reaction times were treated as valid until the end of the inter-stimulus interval, i.e. 1200 ms after stimulus onset. We calculated the median reaction times and conducted Wilcoxon signed-rank tests.

The Wilcoxon tests were conducted for the P200 and the P400 amplitudes and for the median reaction time data with a predefined alpha of 0.05. The resulting *p*-values were corrected for multiple testing with the Holm procedure [38]. The effect size *r*_*es*_ was calculated by dividing the *Z*-score by the square root of the total number of observations [39].

We tested for the influence of the ambiguity level of the temporal context (preceding and subsequent stimuli) on the amplitudes of the S_A_-evoked P200 and P400 ERP components. This was done by comparing conditions S_A_(C_U_) with S_A_(C_A_)_._ The related tests were calculated separately for the S_A_-evoked ERPs and reaction times to the related task from pair one, pair two, and pair three within experimental blocks. This allowed us to investigate the influence of the accumulating perceptual memory, as well as the increasing evidence about stimulus regularity in the temporal context of S_A_ on the reaction times and ERP amplitudes.

## Results from Experiment 2

In Experiment 2, we investigated whether abstract symbolic knowledge about the perceptual future, without sensory history, is sufficient to evoke the effects of sensory quality within the temporal context (P200 and P400) from Exp. 1, or whether the direct perceptual experience of stimulus regularity is necessary. We presented a symbolic announcement of the upcoming conditions S_A_(C_A_) and S_A_(C_U_) at the beginning of each block. Each block consisted of only three stimulus pairs and we analysed S_A_-evoked ERPs from S_A_–pair 1, S_A_–pair 2, and S_A_–pair 3 separately. If the ERP effects found in Experiment 1 are related to processes of predicting the immediate perceptual future, and if the abstract symbolic knowledge about the immediate perceptual future is sufficient to evoke these predictions, we should find effects of sensory quality within the temporal context in the ERPs and in reaction times already in the first stimulus of the first pair. If, however, direct perceptual experience of stimulus regularity is necessary for these effects, we should see earliest evidence for these effects with the first stimulus of the second pair and perhaps a slow build-up of the effect with the third stimulus pair.

### Behavioural data

#### Trial numbers

The average number of valid trials can be found in Table 2. Based on the restriction of a valid response time window from 150 to 1200 ms after stimulus onset, we only had to exclude 0.0006% of all trials (0.003% SD) per participant and condition due to invalid response times. The remaining difference between the average number of all stimulus presentations (Table 2, right column) and the average number of valid trials (Table 2, middle column) between participants and conditions is due to EEG artefacts.

**Table 2:** Number of trials of Experiment 2.

	Average number of valid trials (±SD)	Average number of all stimulus presentations (±SD)
S_A_(C_U_)–pair 1	83 (±17)	109 (±11)
S_A_(C_A_)–pair 1	83 (±17)	110 (±9)
S_A_(C_U_)–pair 2	82 (±19)	108 (±12)
S_A_(C_A_)–pair 2	84 (±20)	109 (±9)
S_A_(C_U_)–pair 3	81 (±19)	105 (±13)
S_A_(C_A_)–pair 3	81 (±20)	107 (±9)

Table 2 displays the average number of valid trials (±SD) in the middle column and the average number of all stimulus presentations (±SD) in the right column, both separately for the experimental conditions. (U = Unambiguous, A = Ambiguous).

#### Reaction times

**Fig 9:**
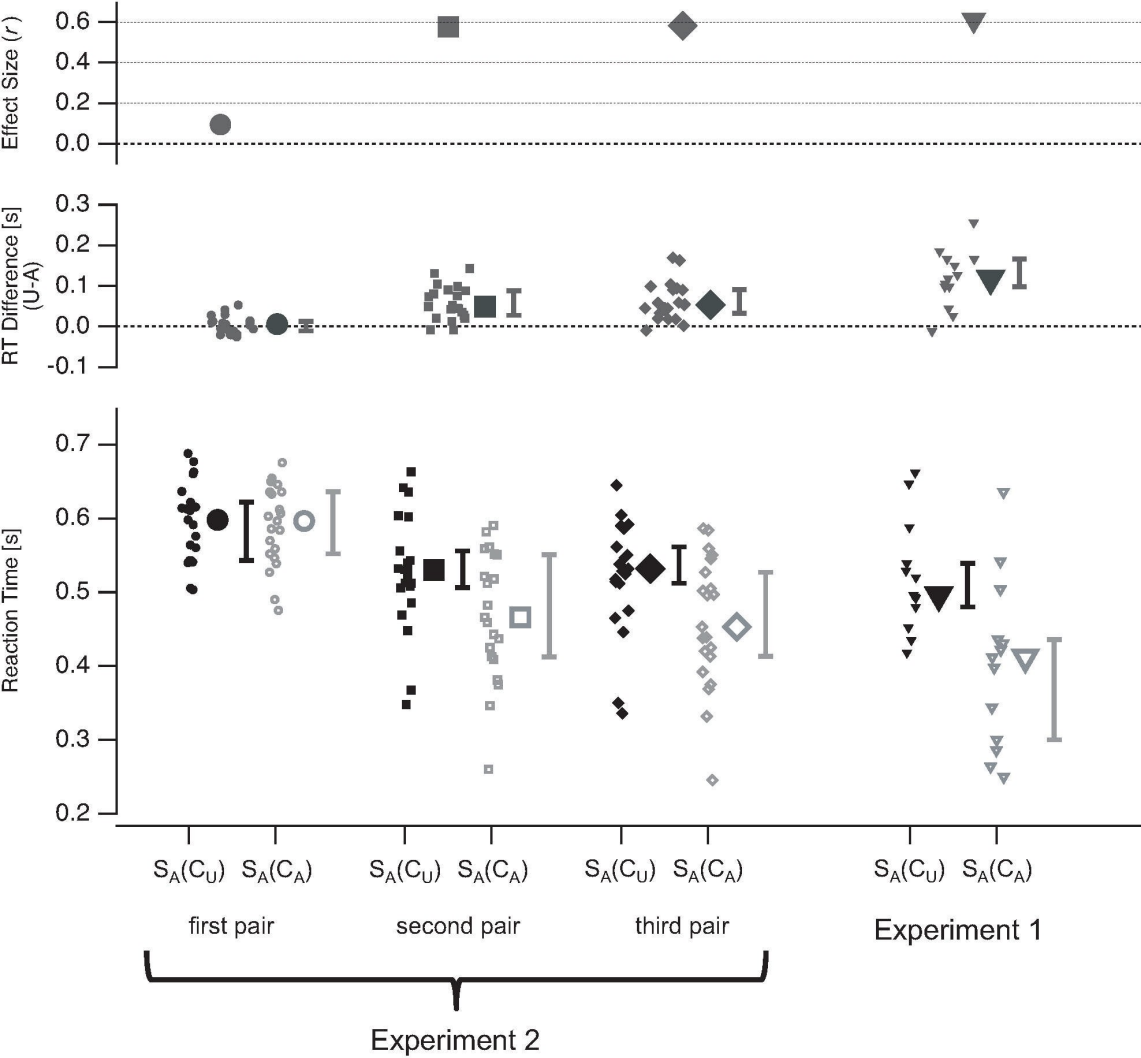
Reaction time results from Experiment 2. Bottom row depicts the median reaction times evoked by a currently observed stimulus separately for the two experimental conditions with unambiguous (black) and ambiguous (grey) stimuli in the temporal context of S_A_. Reaction times are separately shown for the first (circles, first column), second (squares, second column), and third pair (diamond, third column). For comparison, the results from Exp. 1 (triangles) are plotted on the right (fourth column). The data from individual participants are represented with small icons, while the large icons represent the median reaction time data with interquartile ranges (whiskers). In the middle row the median reaction time differences (large icons ± interquartile ranges) between the two conditions (unambiguous temporal context minus ambiguous temporal context) are depicted together with data from the individual participants (small icons). The top row shows the sizes of the reaction time effects (*r*_*es*_). U = Unambiguous, A = Ambiguous.

We found no significant effects of stimulus ambiguity within the temporal context on S_A_-related reaction time in the first pair (S_A_(C_A_)–pair 1 vs. S_A_(C_U_)–pair 1: *Z* = -0.64, *r*_*es*_ = -0.095, *p* = 0.27, see Fig 9). Note that the temporal context of S_A_ from the first pair differs substantially from the temporal contexts of S_A_ in the second and third pair. S_A_ from the first pair was only preceded by a symbolic announcement about the current condition, i.e. the ambiguity levels of the upcoming stimuli. The S_A_ stimuli from the second and the third pair were preceded by lattice stimuli instead of abstract information.

We found significantly longer reaction times for an unambiguous temporal context compared to an ambiguous temporal context in the second (S_A_(C_A_)–pair 2 vs. S_A_(C_U_)–pair 2: *Z* = -3.91, *r*_*es*_ = -0.58, *p* = 2.38e-06) and the third stimulus pair (S_A_(C_A_)–pair 3 vs. S_A_(C_U_)–pair 3: *Z* = -3.95, *r*_*es*_ = -0.58, *p* = 2.86e-06). Note that the reaction times were related to an identification task of the currently seen stimulus S_A_. The temporal context stimuli were completely irrelevant for the execution of this task.

### ERP data

The amplitudes of the S_A_-evoked P200 and P400 show no significant effects of stimulus ambiguity within the temporal context in the first pair (S_A_(C_A_)–pair 1 vs. S_A_(C_U_)–pair 1: P200: *Z* = -0.16, *r*_*es*_ = -0.02, *p* = 0.45; P400: *Z* = -1.16, *r*_*es*_ = -0.17, *p* = 0.42) or in the second pair (S_A_(C_A_)–pair 2 vs. S_A_(C_U_)–pair 2: P200: *Z* = -0.82, *r*_*es*_ = -0.12, *p* = 0.52; P400: *Z* = -0.19, *r*_*es*_ = -0.03, *p* = 0.68). Further, there was no significant effect of stimulus ambiguity within the temporal context for the S_A_-evoked P200 in the third pair (S_A_(C_A_)–pair 3 vs. S_A_(C_U_)–pair 3: P200: *Z* = -1.62, *r*_*es*_ = -0.24, *p* = 0.25;). Interestingly, there was a significant effect of stimulus ambiguity within the temporal context for the S_A_-evoked P400 amplitudes in the third pair (S_A_(C_A_)–pair 3 vs. S_A_(C_U_)–pair 3: P400: *Z* = -2.69, *r*_*es*_ = -0.4, *p* = 0.016).

**Fig 10:**
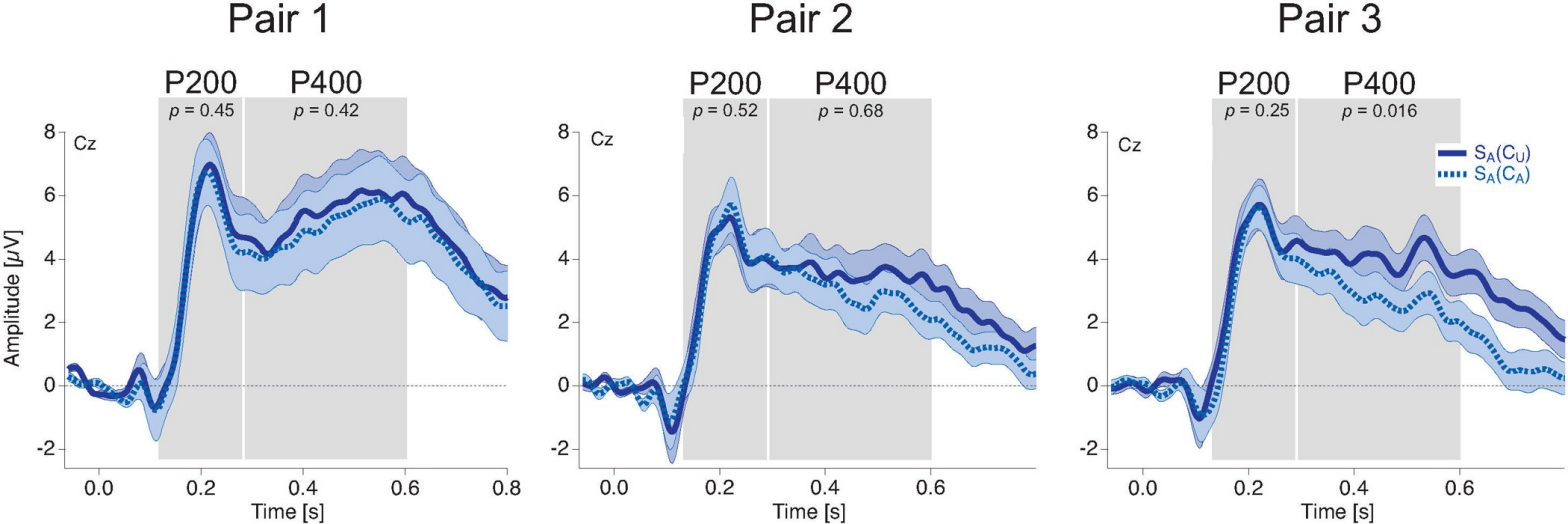
Grand mean ERP results from Experiment 2. Grand means (±SEM) in response to a currently observed stimulus are separately shown for conditions S_A_(C_U_) (dark blue solid lines) and S_A_(C_A_) (light blue dotted lines). S_A_-evoked ERP traces from stimulus pair 1 (left), 2 (middle), and 3 (right) are depicted separately. All traces are displayed for electrode Cz.

**Fig 11:**
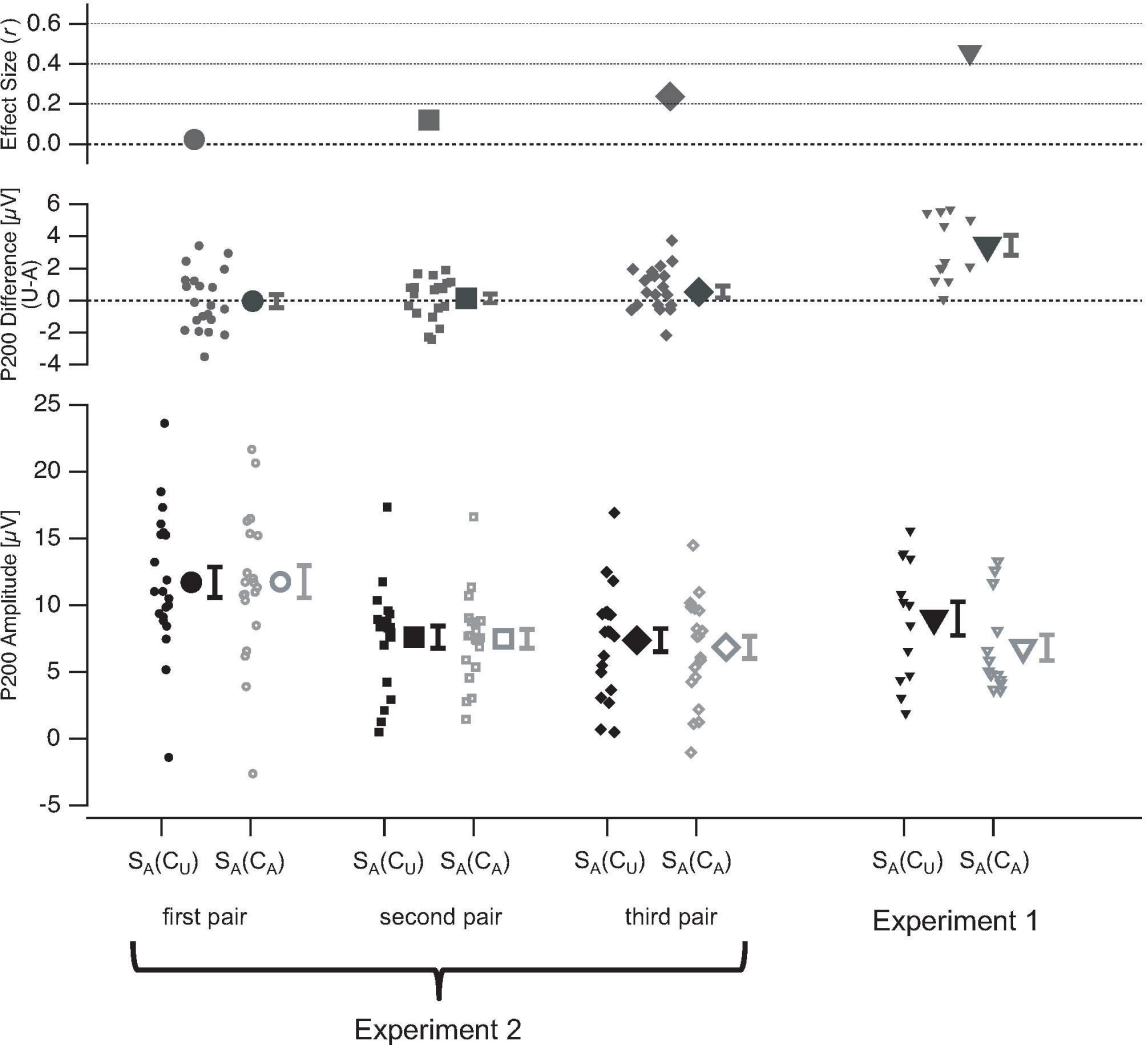
P200 ERP results from Experiment 2. Bottom row depicts the mean amplitudes of the P200 evoked by a currently observed stimulus S_A_ separately for conditions with unambiguous (C_U_, black) and ambiguous (C_A_, grey) stimuli in the temporal context of S_A_. P200 amplitudes are separately shown for S_A_ from the first (circles, first column), second (squares, second column), and third pair (diamond, third column). For comparison, the results from Exp. 1 (triangles) are plotted on the right (fourth column). The data from individual participants are represented with small icons, while the large icons represent the mean amplitudes with SEM (whiskers). In the middle row, the mean ERP differences (large icons ±SEM) between the two conditions (unambiguous temporal context minus ambiguous temporal context) are depicted together with data from the individual participants (small icons). The top row shows the effect size (*r*_*es*_) of the temporal context effects. U = Unambiguous, A = Ambiguous.

**Fig 12:**
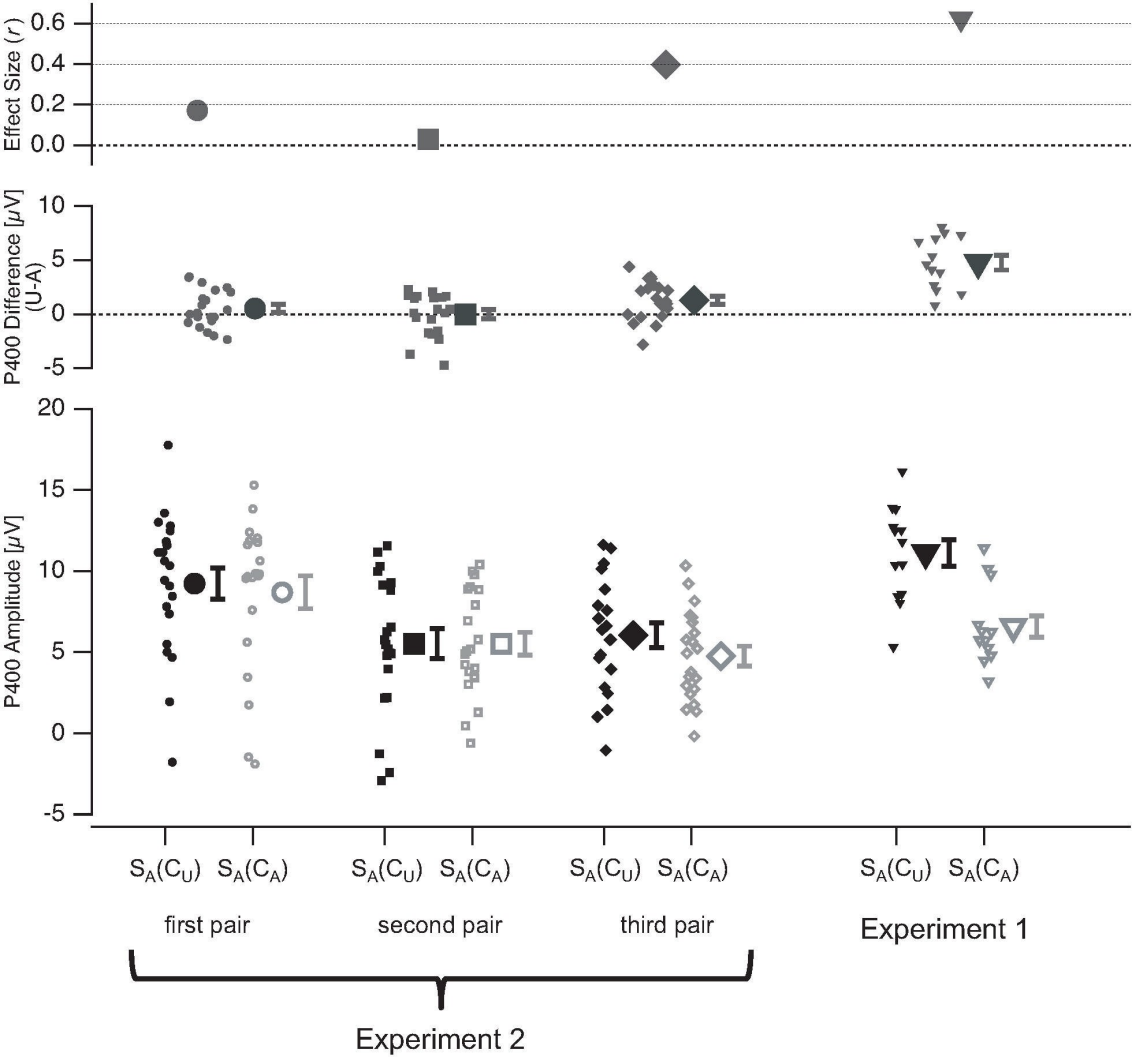
P400 ERP results from Experiment 2. Bottom row depicts the mean amplitudes of the P400 evoked by a currently observed ambiguous stimulus S_A_, separately for conditions with unambiguous (C_U_, black) and ambiguous (C_A_, grey) stimuli in the temporal context. P400 amplitudes are separately shown for the first (circles, first column), second (squares, second column), and third pair (diamond, third column). For comparison, the results from Exp. 1 (triangles) are plotted on the right (fourth column). The data from individual participants are represented with small icons, while the large icons represent the mean amplitudes with SEM (whiskers). In the middle row the mean ERP differences (large icons ±SEM) between the two conditions (unambiguous temporal context minus ambiguous temporal context) are depicted together with data from the individual participants (small icons). The top row shows the effect size (*r*_*es*_) of the temporal context effects. U = Unambiguous, A = Ambiguous.

Fig 10 displays the grand mean ERPs at electrode Cz for the three stimulus pairs separately. Fig 11 shows the summarized results for the three stimulus pairs from Exp. 2 together with the results from Exp. 1 for the P200 and Fig 12 for the P400.

## Summary and discussion of Experiment 2

In Experiment 2, we presented an abstract symbolic announcement before each experimental block and only three stimulus pairs within experimental blocks. We separately analysed ERPs and reaction times from the first, second and third stimulus pair presentations within the blocks. This allowed the comparison between stimulus processing without an immediate perceptual history (first pair) and the slow build-up of a perceptual memory trace across the second and third stimulus pair. The aim of Experiment 2 was to study whether the ERP and reaction times effects of sensory quality within the temporal context are also present when only symbolic knowledge about upcoming temporal context regularities is available (first stimulus pair) and/or whether they build up over accumulating perceptual memory (second and third stimulus pair within experimental blocks). We found no such ERP effects for stimulus S_A_ from the first and second stimulus pair and only a P400 amplitude effect for stimulus S_A_ from the third stimulus pair.

Further, there was no reaction time effect for Task 1 of the first stimulus pair, but similarly strong reaction time effects in the second and the third pair as in Experiment 1.

The results from Experiment 2 indicate that providing only abstract symbolic information about the upcoming stimuli and their ambiguity levels is not sufficient to evoke effects of sensory quality within the temporal context as found in Experiment 1. These effects start to be visible only in the third stimulus pair, i.e. after two exposures to the sensory information and only for the P400 ERP component. Therefore, it can be concluded that the direct sensory experience of regularities is a necessary precondition to evoke the effects of sensory quality within the temporal context found in Experiment 1. This indicates that the abstract symbolic information did not evoke a proper expectation in the participants. Rather, the direct perceptual experience of the stimuli and their regularities must be in the perceptual memory. Although effects of sensory quality within the temporal context for Task 1 reaction times are already present in the second stimulus pair, they are absent for the first pair, again indicating that the direct sensory stimulus experience is a necessary precondition.

## Summary and general discussion

In the present study, we investigated whether the automatic integration of observed regularities across previous percepts and the generation of predictions based on these observed and memorized regularities, affect processing of the sensory present and the execution of a present task. To study this question, we applied a novel experimental paradigm with ambiguous and unambiguous stimulus variants and investigated two ambiguity-sensitive ERP components (P200 and P400). We used stimulus ambiguity to manipulate the reliability of the perceptual history and the predictions about the perceptual future.

We found that the amplitudes of P200 and P400 ERPs evoked by identical lattice stimuli differ as a function of the temporal context, i.e. the ambiguity level of a preceding stimulus (S2 from the previous pair) and the expected ambiguity level of a subsequent stimulus (S2 from the current pair). Similarly, reaction times from a stimulus-related Task 1 differ as a function of the ambiguity level of the stimuli within the temporal context, even though they were irrelevant for the execution of this task.

In the following, we will first discuss general limitations of the current study. We will then describe previous findings of P200 and P400 ERP components, similar to the ones found in the present study. Next, we discuss whether our results reflect "footprints" from the perceptual past or rather mechanisms underlying predictions of the perceptual future, or both. Finally, we will speculate about the specific functional roles of the identified ERP effects and provide a possible explanation for the differing pattern of reaction time effects.

### Limitations of the current study

In Experiment 1, thirteen participants were measured. We conducted a power analysis [45] on the basis of previous results [26,27], which indicated 12 participants as sufficient. Even though the previously found exceptionally large P200 and P400 amplitude effects related to stimulus ambiguity [26,27,31] suggested this small number of participants, a larger number of participants in a follow-up replication of the present study may confirm the present findings and even reveal more subtle effects.

Our current EEG setup contains only 32 electrode channels. For future attempts to determine the brain sources underlying the reported effects, one should use a setting with 64 or even 128 electrodes, combined with anatomical MRI scans. These two extensions would decrease the well-known inverse problem in EEG, when trying to identify sources [46,47].

### P200 and P400 in the literature

Only a few studies report a positive deflection with a fronto-central distribution 200 ms after stimulus onset, as shown in the current study. Similar P200 ERP components were found during feature detection across visual dimensions [48], modality-independent emotional salience [49], and the match of sensory input with memory contents [11]. The latter finding is in line with our current finding of a P200 amplitude modulation, which is dependent on information from the temporal context. Perri et al. [50] found frontal components (pP1, pP2) in the P200 time-range related to decision-making. These ERP components originate from the anterior insula and reveal larger amplitudes in response to complex compared to simple stimuli. The present P200 findings show an opposite pattern, i.e. larger ERP amplitudes in response to easy perceptual decisions (unambiguous stimuli) and smaller ERP amplitudes in response to difficult/uncertain perceptual decisions (ambiguous stimuli). It may be very interesting to systematically compare similarities and differences in the paradigms and results of the study by Perri et al. and the current experiments in a follow-up study, which then may include source analysis, in a way suggested above.

The positivity 400 ms after stimulus onset with its central distribution resembles the well-known P300 ERP component (specifically P3b [42]). The P300 is typically reported in "oddball paradigms" evoked by infrequent and task-relevant stimuli. The P300 latency is found to be negatively correlated with reaction times (for reviews see [43,44]). In the current study, we aimed at excluding typical oddball situations as much as possible in order to avoid P300 contributions to our results. The present P400 may still share some neural mechanisms with the P300 but cannot be completely reduced to it, at least because of the obvious differences in the respective characteristics. This issue has been discussed in more detail in a recent publication of Kornmeier et al. [27].

### Do the present findings reflect "footprints" from the past or predicting the future—or both?

Our findings indicate that the amplitude modulations of the P200 and P400 ERP components evoked by one and the same stimulus are based on different sensory qualities within the temporal contexts. One interesting question is now, whether these amplitude effects result from predictions about the future or whether they are "footprints" from the perceptual past–or whether both factors play a role?

Effects of the perceptual history on early perceptual processing of a current stimulus are well known from the literature. The immediate perceptual history can have both facilitating (serial dependence) and inhibitory (adaptation) effects on the perceptual outcome during observation of ambiguous figures [9,10,13–15,51]. Further examples are motion aftereffects [52], contrast aftereffects [18], or repetition suppression [53]. Moreover, both serial dependence and adaptation effects can be found at different levels along the perceptual processing chain, up to the processing of emotional contents of faces and even beyond [9,54,55].

The amplitude differences of the P200 and P400, as found in the present study, may be simply caused by low-level influences from the immediate past rather than reflecting predictions about stimuli in the immediate future. In particular, the results from Experiment 2 point in this direction: Given our experimental design, stimulus S1 from the first of three stimulus pairs and the related Task 1 have no influential immediate perceptual history (see Methods above). Accordingly, the amplitudes of the P200 (Fig 11) and P400 (Fig 12) evoked by the first stimulus of the first stimulus pair and the corresponding task-related reaction times (Fig 9) do not differ between conditions. The influence from the past seems to build up over time: we found reaction time effects as early as the second stimulus pair and also for the third pair. We found P400 amplitude effects for the third pair, which has a perceptual history of two preceding stimulus pairs, but no significant effects for the P200.

On the other hand, it is also possible that the current amplitude and reaction time modulations reflect processes underlying the generation of predictions about the future rather than footprints from the past. A necessary precondition for reliable predictions about the future is the identification of reliable statistics in the past. Having this in mind, the results from Experiment 2 are also compatible with the predicting approach: if there is no history of regularities, there will also be no reliable source for the generation of a prediction and thus, neither a difference in ERP amplitudes nor in reaction times, as found for the first stimulus pair in Experiment 2. Further, with an accumulating perceptual history (second and third stimulus pair) including mounting evidence for regularities, predictions can be generated and become increasingly reliable, resulting in ERP amplitude and reaction time differences between conditions. We found P400 amplitude effects for the third stimulus pair and observed in Fig 11 a weak but not yet significant tendency for a P200 amplitude difference with this third stimulus pair. Interesting in this context is a study by Jazayeri and Shadlen [56]. They demonstrated that if stimuli are drawn from a certain distribution, perception of a current stimulus is biased towards the mean of the distribution that the stimulus originates from, and that response behaviour is best explained by a Bayesian observer model. Of course, this is only possible if the observer relies on a certain type of statistics across a certain time window of repeated presentations of stimuli from this specific distribution. One interesting question is, how many stimuli from such a distribution need to be presented in order to get a reliable estimate of the distribution’s mean. Correspondingly, it would be interesting to extend our Experiment 2 by adding more stimulus pairs to one experimental block and see at which point effect sizes as found in Experiment 1 are reached. Such a follow-up study is on our agenda.

Jazayeri and Shadlen [56] investigated perceptual un/certainty by varying temporal aspects of the stimuli, i.e. presenting stimuli for different durations. The current study might add to this line of research in that it also investigates perceptual un/certainty but here this is introduced through the sensory quality of the stimulus instead of its temporal extend.

The considerations above raise the fundamental question whether memory and prediction effects are at all experimentally separable or whether they are inextricably entangled. Current predictive coding approaches [24] assume numerous cycles of generating and evaluating predictions along the (hierarchical) chain from early sensory to cognitive processing [57]. Effects of adaptation and/or serial dependence, typically labelled as low-level sensory effects [58], may thus be exploited during the prediction process and have been discussed to involve predictive properties [59]. One can therefore inversely ask the question whether low-level memory effects, as mentioned above, can occur without influencing the prediction of future sensory input.

### Effects of sensory quality within the temporal context on reaction times

The reasoning above may also explain longer reaction times related to a currently observed stimulus if an ambiguous future stimulus is expected, compared to the expectation of an unambiguous future stimulus. The problem with this, initially intuitive, explanation of the reaction time effects is that it only fits to those conditions with an *unambiguous* currently observed stimulus S1. For an *ambiguous* current stimulus, we found the opposite pattern, i.e. longer reaction times with an unambiguous temporal context.

This finding seems counter-intuitive at first sight, but makes much more sense if we look at it from a different point of view: There is *perceptual continuity over time* in the case of S_A_(C_A_), i.e. if both the currently observed stimulus and its temporal context are ambiguous. Similarly, there is perceptual continuity over time in the case of S_U_(C_U_), i.e. if both the currently observed stimulus and its temporal context are unambiguous. In contrast, there is *perceptual discontinuity over time* in the case of S_A_(C_U_), i.e. if the currently observed stimulus is ambiguous and its temporal context is unambiguous. There is also perceptual discontinuity over time in the case of S_U_(C_A_). Reaction times were consistently shorter in the perceptual continuity case compared to a perceptual discontinuity case. The reaction time effect thus may not reflect the expected quality of a stimulus but, in contrast, the expected perceptual continuity or discontinuity–concerning ambiguity levels–between the currently observed stimulus and its temporal context. This interpretation stands in line with behavioural findings from task-switching paradigms [60]. They show slower reaction times when the task switches from one stimulus to the next, and faster reaction times when the tasks stay the same. However, the ERP effects found in task-switching paradigms [60] are different from our findings. This indicates that the ERP effects reflect a different processing step in the current study than in studies only dealing with task switching and not ambiguity level switching.

To sum up, our results indicate that the perceptual system exploits regularities from the immediate perceptual past in order to generate predictions about the expected sensory quality of a future stimulus in at least two steps, reflected by the P200 and P400 ERP components. Separately, automatic predictions are generated about perceptual continuity, i.e. whether the given sensory quality of a stimulus at time point t_1_ is expected to continue to another stimulus at time point t_2_. An expected change of sensory quality may require the pre-activation of additional neural resources in order to be prepared for an expected larger environmental change. This pre-activation may increase reaction times related to the execution of a current task by about 100 ms, even though this task is restricted to the sensory evidence from the current stimulus, while perceptual past and expected perceptual future are irrelevant. Of course, this interpretation is speculative and needs to be further confirmed or even disproved in future studies.

### What do the present findings tell us about the integration of information from the temporal context?

The available sensory information is noisy, incomplete and, to varying degrees, ambiguous. Thus, finding the most appropriate perceptual interpretation as quickly and as efficiently as possible was, most probably, a critical factor during the evolution of perception. This is known as the perceptual inference problem [1] and has the consequences that we exploit even tiniest bits of stimulus [40] and contextual information [61] in order to resolve it. Adaptation [62,63] and serial dependence [14–16] are examples of how past temporal regularities in the sensory environment influence current percepts. Predictions about the immediate future based on current percepts and identified regularities in the past, can facilitate and optimize the perceptual process, because typically our environment does not change fundamentally from one moment to the next. Thus, several of the previous arduously created perceptual concepts can simply be kept for the next perceptual moment [64].

Interestingly, even though predictions obviously influence conscious experience, they seem to be generated automatically and neither awareness nor task-relevance of, or attention to the predicted stimulus seem to be necessary preconditions [e.g. 65,66].

Most studies on predictive coding focused on how predictions facilitate perception [20–22]. Only a few studies focused on neural correlates of *generating predictions* at a time point t_1_ concerning the expected sensory information at a time point t_2_ (few examples are fMRI measurements in humans [65] and voltage sensitive dye measurements in ferrets [67]). The current results can be interpreted as evidence for EEG and behavioural measures of how making predictions about the future, based on regularities in the past, affects perceptual processing of a present stimulus and execution of a present task. We found larger amplitudes of two ERP components evoked by the same stimuli when an unambiguous future stimulus can be predicted from the temporal context than when an ambiguous future stimulus can be predicted (effect sizes between 0.24 and 0.62).

Also noteworthy is the observation that the sensory quality within the temporal context also modulates reaction times of the present Task 1, even though the sensory quality within the temporal context is completely irrelevant for the execution of this task. This is further evidence for the inevitability and automaticity of integration of information from the temporal context.

The present results are threefold and may reflect three processing steps. The first step is indicated by the modulation of the fronto-central P200. The second step follows 200 ms later, as indicated by the modulation of the centro-parietal P400 and the deviating pattern of reaction time results indicate a different third step. Both ERP effects point in the same direction (larger amplitudes if stimuli in the temporal context were unambiguous compared to ambiguous), but the results indicate small differences between the P200 and the P400 amplitude effects. Particularly, the results from Experiment 1 show that the ambiguity level of the temporal context of a currently observed stimulus affects the P400 amplitude, irrespective of the ambiguity level of the observed stimulus itself. However, we only see P200 effects of sensory quality within the temporal context if the currently observed stimulus is ambiguous. Further, although the functional difference between processes underlying the two ERP components is currently not entirely clear, recent evidence from our lab indicates that the fronto-central P200 component reflects—at least partly—the reactivation of memory traces during comparison of present with previous perceptual interpretations [68,69]. The absence of the P200 effects of sensory quality within the temporal context during observation of an unambiguous current stimulus may be a simple ceiling effect: The amplitude of the P200 evoked by an unambiguous currently observed stimulus S1 is larger than the amplitude of the P200 evoked by an ambiguous stimulus S1 (compare dashed traces in Fig 6A and 6C). The amplitude of the P200 evoked by an unambiguous S1 may already be at such a high level that it cannot further increase due to physiological and/or brain anatomy reasons. On the other hand, it may also be possible that in a situation when an unambiguous stimulus S1 is currently observed, i.e. when high quality sensory evidence is present, a possible contribution of the working memory may be reduced and a potentially smaller P200 effect of sensory quality within the temporal context may become insignificant.

With the simplified explanation of the effects of sensory quality within the temporal context on the processing of a current stimulus reported in this study, one could assume that–based on reactivated information from perceptual memory–large amplitudes reflect the expectation of an unambiguous and thus, easy-to-process future stimulus. This may therefore serve as a kind of go-signal, affecting both the current task (i.e. faster reaction times), and the future perceptual process. Expecting an ambiguous, thus unclear future, in contrast, may cause an inhibition of the go-signal (resulting in smaller ERP amplitudes). Furthermore, expecting a discontinuity in the visual flow over time may result in a more careful execution of actions in the present. All of this is currently a very speculative explanation of our results and far away from a well-founded theoretical framework. Of course, further experimental steps are necessary to get a clearer picture and to confirm or disconfirm these speculative interpretations.

## Conclusion and outlook

One strategy to make perception metabolically more efficient, more reliable and faster may include accounting for stimulus regularities in the past to anticipate the immediate future. Predictive strategies are thus powerful contributions to the resolution of the perceptual inference problem [1]. The current findings show that information from the temporal context strongly modulate perceptual processes in a highly automatic manner, even if those temporal context stimuli are outside the focus of attention and irrelevant for a given task. It seems as if the information from the temporal context is always integrated into the current percept and we cannot avoid doing so. Further evidence for such automaticity comes from the observation that the direct experiences of perceptual regularities in the past are necessary preconditions for the current effects to occur. Symbolic announcements at a higher cognitive level alone are not sufficient, as found in Experiment 2.

Previous studies about influences of past events and prediction mechanisms during perception used unambiguous and clearly visible stimuli. Typical studies on prediction mechanisms compared frequently presented and thus highly predictive stimuli with rare unpredictable stimuli. Stimulus frequency, not stimulus quality, was the critical factor. The current study used stimulus quality, namely stimulus ambiguity, as the critical factor. In our study, the compared perceptual situations were identical concerning stimulus frequency but different concerning perceptual reliability. The different paradigms may have lead to differing results. An interesting next step may be to bridge the gap between the different approaches and to extend the given theoretical framework in a follow-up step by incorporating the differing results.

The current findings indicate that the sensory quality within the temporal context can influence the present and slow down a current task. Importantly, this is the case even when the immediate past and the immediate future are irrelevant for the execution of this current task. Therefore, integrating information from the temporal context may, in certain situations, have impeding effects on how we see the present and how (fast) we act at a present moment. Everyone is familiar with situations where the anticipation of an unpleasant future inhibits us mentally or makes us less motivated during an unrelated current task. Depression may be an extreme case of such a scenario. The current results highlight lower-level perceptual states and mechanisms whose characteristics potentially parallel those of such higher-level mental states and mechanisms. Depression is typically regarded as a higher-level psychiatric disorder. Interestingly, recent evidence indicates that also lower-level visual processing steps are affected [e.g. 70]. It may be interesting to apply the current paradigm to patients with depression and see whether they show an altered pattern of effects of sensory quality within the temporal context. This is one of our next steps on the agenda.

A better understanding of the mechanisms underlying the automatic integration of the temporal context for the generation of predictions may thus help to understand basic principles of perception. At the same time, it may also help to better understand basic principles of higher-level mental states and mental disorders.

## Supporting information

S1 File Result tables of correlations between EEG data, reaction time data, and reversal rates. (DOCX)
