# Supplementary material for: Using the perceptual past to predict the perceptual future influences the perceived present – A novel ERP paradigm
Source: PLoS One. 2020 Sep 1;15(9):e0237663. doi: 10.1371/journal.pone.0237663 (PMC7462302; doi:10.1371/journal.pone.0237663)
Supplement: S1 File — (DOCX) [file pone.0237663.s001.docx]

**S1 File. Result Tables of correlations between EEG data, reaction time data, and reversal rates.**

**Table A. Correlation between EEG data (P200, P400) and reaction time data.**

|  | Condition | Pearson correlation coefficient | *p*-value |
| --- | --- | --- | --- |
| P200 | S_A_(C_A_) | -0.346 (-0.07) | 0.25 (0.59) |
|  | S_U_(C_U_) | 0.33 (0.1) | 0.14 (0.37) |
|  | S_U_(C_A_) | -0.29 (-0.35) | 0.32 (0.24) |
|  | S_U_(C_U_) | 0.17 (-0.27) | 0.29 (0.36) |
| P400 | S_A_(C_A_) | -0.36 (-0.16) | 0.23 (0.61) |
|  | S_U_(C_U_) | 0.18 (0.14) | 0.28 (0.32) |
|  | S_U_(C_A_) | -0.002 (-0.29) | 0.5 (0.35) |
|  | S_U_(C_U_) | 0.03 (-0.39) | 0.46 (0.19) |

Table A displays the Pearson correlation coefficients and corresponding *p*-values for normalized (and non-normalized) data between the EEG data (P200 and P400 amplitudes) and the median reaction time data. No significant correlations were found.

**Table B. Correlation between EEG data (P200, P400) and reversal rates.**

|  | Condition | Pearson correlation coefficient | *p*-value |
| --- | --- | --- | --- |
| P200 | S_A_(C_A_) | -0.03 (0.27) | 0.54 (0.19) |
|  | S_U_(C_U_) | 0.34 (0.25) | 0.13 (0.21) |
|  | S_U_(C_A_) | 0.12 (0.03) | 0.35 (0.46) |
|  | S_U_(C_U_) | 0.41 (0.09) | 0.08 (0.38) |
| P400 | S_A_(C_A_) | -0.12 (0.04) | 0.66 (0.45) |
|  | S_U_(C_U_) | 0.19 (0.19) | 0.26 (0.26) |
|  | S_U_(C_A_) | -0.19 (-0.49) | 0.53 (0.08) |
|  | S_U_(C_U_) | -0.16 (-0.24) | 0.6 (0.43) |

Table B displays the Pearson correlation coefficients and corresponding *p*-values for normalized (and non-normalized) data between the EEG data (P200 and P400 amplitudes) and the reversal rates. No significant correlations were found.

**Table C. Correlation between reversal rates and reaction time data.**

| Condition | Pearson correlation coefficient | *p*-value |
| --- | --- | --- |
| S_A_(C_A_) | 0.61 (0.61) | 0.01 (0.01) |
| S_U_(C_U_) | 0.3 (0.39) | 0.15 (0.1) |
| S_U_(C_A_) | 0.04 (0.51) | 0.45 (0.04) |
| S_U_(C_U_) | 0.46 (-0.18) | 0.06 (0.56) |

Table C displays the Pearson correlation coefficients and corresponding *p*-values for normalized (and non-normalized) data between the reversal rates and the median reaction time data. Only in condition S_A_(C_A_) a significant correlation was found.
